# Accurate estimation of 5-methylcytosine in mammalian mitochondrial DNA

**DOI:** 10.1038/s41598-018-24251-z

**Published:** 2018-04-11

**Authors:** Shigeru Matsuda, Takehiro Yasukawa, Yuriko Sakaguchi, Kenji Ichiyanagi, Motoko Unoki, Kazuhito Gotoh, Kei Fukuda, Hiroyuki Sasaki, Tsutomu Suzuki, Dongchon Kang

**Affiliations:** 1 Department of Clinical Chemistry and Laboratory Medicine, Graduate School of Medical Sciences, Kyushu University, 3-1-1 Maidashi, Higashi-ku Fukuoka, 812-8582 Japan; 2 Department of Chemistry and Biotechnology, Graduate School of Engineering, The University of Tokyo, 7-3-1, Hongo, Bunkyo-ku Tokyo, 113-8656 Japan; 3 Division of Epigenomics and Development, Medical Institute of Bioregulation, Kyushu University, 3-1-1 Maidashi, Higashi-ku Fukuoka, 812-8582 Japan; 4 Present Address: Laboratory of Genome and Epigenome Dynamics, Department of Applied Molecular Biosciences, Graduate School of Bioagricultural Sciences, Nagoya University, Furo-cho, Chikusa-ku Nagoya, 464-8601 Japan; 5 Present Address: Cellular Memory Laboratory, RIKEN, 2-1 Hirosawa, Wako Saitama, 351-0198 Japan

## Abstract

Whilst 5-methylcytosine (5mC) is a major epigenetic mark in the nuclear DNA in mammals, whether or not mitochondrial DNA (mtDNA) receives 5mC modification remains controversial. Herein, we exhaustively analysed mouse mtDNA using three methods that are based upon different principles for detecting 5mC. Next-generation bisulfite sequencing did not give any significant signatures of methylation in mtDNAs of liver, brain and embryonic stem cells (ESCs). Also, treatment with methylated cytosine-sensitive endonuclease McrBC resulted in no substantial decrease of mtDNA band intensities in Southern hybridisation. Furthermore, mass spectrometric nucleoside analyses of highly purified liver mtDNA preparations did not detect 5-methyldeoxycytidine at the levels found in the nuclear DNA but at a range of only 0.3–0.5% of deoxycytidine. Taken together, we propose that 5mC is not present at any specific region(s) of mtDNA and that levels of the methylated cytosine are fairly low, provided the modification occurs. It is thus unlikely that 5mC plays a universal role in mtDNA gene expression or mitochondrial metabolism.

## Introduction

Methylation of the fifth carbon of cytosine residues in mammalian nuclear DNA plays crucial epigenetic regulatory roles in diverse processes, including differentiation and cellular reprogramming^1,2^. In human genomes, about 4% of cytosines are methylated^3^, and most of these are present at CG dinucleotide sequences, with a frequency of about 80% on cytosines of CG sequences.

Mammalian mitochondria contain multicopy circular genome of approximately 16 kb, called mtDNA. The two strands of mtDNA are distinguished on the basis of nucleotide compositions and are referred to as light and heavy strands (L and H strands, respectively). mtDNA encodes 13 subunits of the oxidative phosphorylation complexes and 2 ribosomal RNAs and 22 transfer RNAs for translation of these subunits in mitochondria. The importance of mtDNA can be recognised by the fact that mutations in mtDNA and mtDNA depletion are associated with human pathology. Such abnormalities impair the oxidative phosphorylation system and result in a variety of clinical symptoms that frequently affect the neuronal system and cardiac and skeletal muscle^4–6^.

Currently, there is no consensus on methylation of mammalian mtDNA. Several early studies concluded that mtDNA contains either no or very low amounts of 5mC in cultured cells and animal liver tissues^7–11^. Moreover, no methylation of mtDNA was detected in cancer cell lines or tissues from cancer patients^12^. In contrast to these reports, other early studies proposed that the percentages of 5mC are higher in mtDNA than in nuclear DNA in animal liver and heart tissues^13,14^. Then, two recent studies made strong proposition that substantial amounts of the methylated cytosines were detected from mtDNA in human and mouse cultured cells; Infantino *et al*.^15^ reported detection of 5-methyldeoxycytidine (5mC-deoxyribose) in mass spectrometry analysis of mtDNA and Shock *et al*.^16^ proposed that 5mC was observed in mtDNA using methylated DNA immunoprecipitation. Further to these reports methylated cytosines were suggested to be present in mtDNA in various materials^17–25^. However, degrees and patterns of methylation were not fully clarified by such reports, and even confusion/doubt on mtDNA methylation could be raised when conflicting data about patterns of methylation were seen among the reports. No integrated picture of mtDNA methylation can thus be drawn from them. Furthermore, another recent investigation^26^ concluded the absence of appreciable methylation in mtDNA in human cells using bisulfite sequencing^27,28^. Further to the ambiguity of mtDNA methylation, controversial claims were made regarding whether or not DNA methyltransferases, DNMT1, DNMT3A and DNMT3B, which are responsible for nuclear cytosine methylation, are also present in mitochondria (for review see Maresca *et al*.^29^), adding more confusion to the issue of mtDNA methylation.

Such a blur situation as well as potential importance of mtDNA methylation and clinical relevance of its disturbance, if mtDNA is indeed methylated, prompted us to thoroughly investigate 5mC in mammalian mtDNA. To this end, we used bisulfite sequencing, McrBC digestion analyses and liquid chromatography mass spectrometry (LC/MS), which are distinctly differing methods for detecting 5mC. Furthermore, great care was taken to sample preparations, experimental manipulations and data analyses. Bisulfite sequencing was performed using mitochondrial nucleic acid (mtNA) fractions to obtain reads derived from mtDNA exclusively, and robust control DNAs were used to ensure that unmodified cytosines in mtDNA were not falsely identified as 5mC. Under these experimental conditions, we analysed mtDNAs from mouse ESCs and from mouse liver and brain tissues. We also examined 5mC in mtDNA of ESCs and tissues using the bacterial enzyme McrBC, which cleaves DNA containing 5mC with relaxed sequence specificity^30^, offering a powerful tool for detecting 5mC in DNA. Finally, we examined the presence of 5mC in mtDNA samples from liver tissues by LC/MS after preparing samples with an extensive purification procedure. Our exhaustive investigation revealed challenging points in studying the methylation of mtDNA and led us to propose the real status of 5mC in mtDNA.

## Results

### Whole genome bisulfite sequencing (WGBS) data show peculiar patterns of methylation in ESC mtDNA

Bisulfite sequencing relies on bisulfite conversion reaction in which unmodified cytosines in DNA strands are converted to uracils while 5mCs remain unaffected. Subsequent primer extension step(s) and/or DNA amplification step introduces thymine to the positions of uracil and cytosine to those of 5mC. The resulting DNA is then sequenced and the extent of methylation at each cytosine position is estimated from the extent of ‘unconversion’^27,28^.

WGBS combines bisulfite conversion and next-generation sequencing, and can be used to obtain information of the methylation status of a whole genome^31^. Because WGBS samples should contain all DNA from cells, as a preliminary study we examined the status of mtDNA using published WGBS metadata. Reads from mtDNA were extracted from them and aligned to reference mtDNA sequences. Then, to exclude the possibility of mapping H strand-derived reads to L strand and *vice versa*, if a read contains a G to A substitution and the average unconversion rate of cytosines in non-CG sequences in the read was ≥70%, such a read was discarded. After filtration, mtDNA methylation was estimated. Whereas effectively no signature of methylation was observed in mtDNA of tissues from mouse organs and a couple of human samples, reads from mtDNA of some samples, such as mouse ESCs, contained significant amounts of unconverted cytosines at both CG and non-CG sites, with strong preference for L strands (Supplementary Fig. S1 and Supplementary Table S1). The peculiar patterns prompted us to prepare samples from mouse ESCs and tissues by ourselves and investigate mtDNA methylation.

### Bisulfite sequencing of a selected region of ESC mtDNA did not detect 5mC

**Figure 1:**
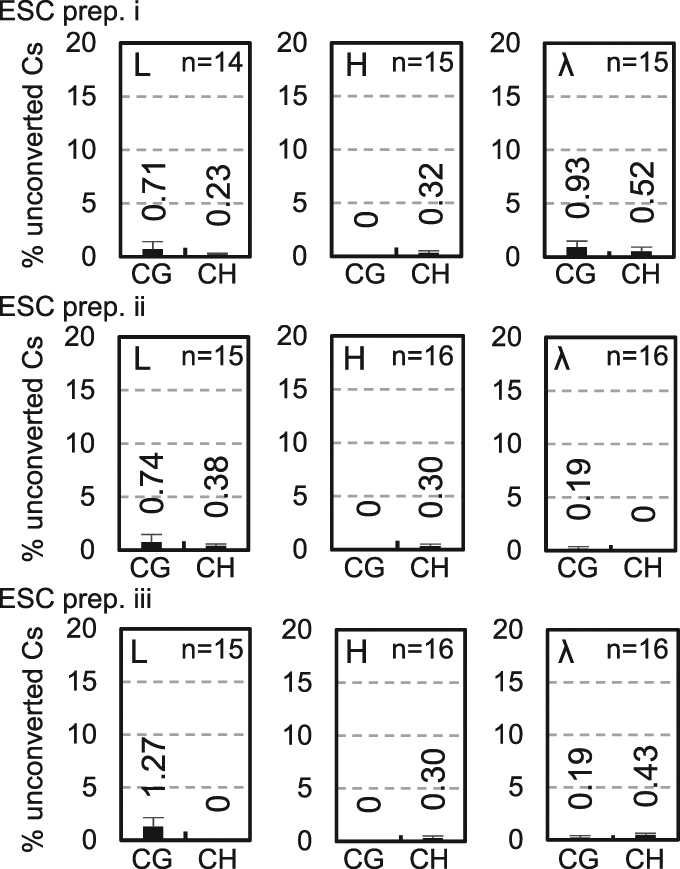
Bisulfite sequencing of cytochrome *b* gene region of ESC mtDNA. Three independent preparations of embryonic stem cell (ESC) mtDNA (ESC preps. i–iii) were examined by bisulfite sequencing. After bisulfite conversion, L and H strands of the cytochrome *b* gene region were PCR-amplified and cloned into T-vectors. Average unconversion rates of cytosines at CG sequences (CG) and those at non-CG sequences [CH (H = A, G and T)] in the cloned fragments were obtained from sequencing data for inserts of each plasmid clone, and means of average rates were calculated with SEM for L strands (L) and H strands (H). Data are presented as percentages. A region of control λDNA^−mC^ was also analysed to confirm conversion efficiency of the reactions (λ). n indicates the number of clones analysed in each category.

Because the cyto- chrome *b* (cyt*b*) gene region in ESC mtDNA showed a strong signature of unconverted cytosines in the above analyses (data not shown), we analysed this region using bisulfite sequencing with a conventional cycle sequencing method. mtNA that was prepared from highly purified mitochondria [sucrose density-gradient centrifugation (SDGC)-purified, nuclease-treated mitochondria, N-Mito; see below and Methods for details] from ESCs was used as the starting material to exclude the possibility of picking up numts (nuclear DNA sequences of mitochondrial origin). Because bisulfite conversions occur on cytosine residues that are not base-paired, it is crucial to denature DNA prior to the conversion step. mtNA was treated with the restriction enzyme DraI to linearise and fragment mtDNA (Supplementary Fig. S2a) to ensure denaturation. Subsequently, mtNA was spiked with methylcytosine-free lambda DNA (λDNA^−mC^), and was then heat-denatured and subjected to bisulfite conversion. Alkaline denaturation was avoided as mtDNA contains ribonucleotides in the strands^32,33^. L and H strands in the cyt*b* gene region were then respectively amplified from bisulfite-converted templates using strand-specific primers. The resulting PCR fragments were cloned into T vectors and numerous clones were sequenced. The obtained sequences were then aligned with reference sequences and methylated cytosine levels were calculated in each fragment. A λDNA region was also amplified from the same bisulfite-treated samples, cloned and sequenced as an internal control. Under the conditions in which cytosines in λDNA^−mC^ were converted with >99% conversion rate, cytosines in both strands of the cyt*b* region of ESC mtDNA were effectively completely converted in both CG and non-CG [CH (H = A, G and T)] sequences (>98.5%) (Fig. 1).

### Next-generation bisulfite sequencing of mtDNA did not reveal substantial methylation signatures

Because initial analyses of published WGBS data and cycle sequencing-based bisulfite sequencing of the cyt*b* region of ESC mtDNA gave inconsistent results, we decided to examine the entire mtDNA of ESCs (3 independent preparations) and those of liver and brain (2 independent preparations) using a next generation sequencer.

We used mtNA from highly purified mitochondria (N-Mito) as the starting material to exclusively capture mtDNA-derived fragments for deep sequencing. In these experiments, mtNA was treated with the restriction enzyme BglII to linearise mtDNA and was then spiked with λDNA^−mC^. It is a nearly 50 kb linear DNA, which is much larger than mtDNA. After heat denaturation, bisulfite conversion was performed for 5, 15, 40, 60 and 90 min to examine the efficiency of conversion. For comparison, a 16,291 bp long ‘synthetic mtDNA’, which mimics BglII-digested native mtDNA (Supplementary Fig. S2a), was generated by PCR using native mtDNA (16,299 bp) as a template and was used as an authentic 5mC-free mtDNA. DNA libraries were then constructed from 24 samples (Table 1) using post-bisulfite adapter tagging (PBAT) method^34^ (Supplementary Fig. S2b) and were subjected to multiplex deep sequencing using an Illumina MiSeq. A total of 11 million reads (0.45 million reads per library) was yielded. Using these reads (150 bp), the methylation states were analysed using Bismark^35^, producing the data set with a median sequence depth per cytosine residue of 96.

**Table 1:** Sample list of MiSeq analysis.

Index No.	Samples	Duration (min)	Abbreviation
1	Liver mtNA prep - 1	5	L1_05
2	Liver mtNA prep - 1	15	L1_15
3	Liver mtNA prep - 1	40	L1_40
4	Liver mtNA prep − 1	60	L1_60
5	Liver mtNA prep − 2	60	L2_60
6	Brain mtNA prep - 1	5	B1_05
7	Brain mtNA prep - 1	15	B1_15
8	Brain mtNA prep - 1	40	B1_40
9	Brain mtNA prep - 1	60	B1_60
10	Brain mtNA prep - 2	60	B2_60
11	Synthetic mtDNA	5	S1_05
12	Synthetic mtDNA	15	S1_15
13	Synthetic mtDNA	40	S1_40
14	Synthetic mtDNA	60	S1_60
15	ESCs mtNA prep - 1	5	E1_05
16	ESCs mtNA prep - 1	15	E1_15
18	ESCs mtNA prep - 1	40	E1_40
19	ESCs mtNA prep - 1	60	E1_60
20	ESCs mtNA prep - 2	5	E2_05
21	ESCs mtNA prep - 2	15	E2_15
22	ESCs mtNA prep - 2	40	E2_40
23	ESCs mtNA prep - 2	60	E2_60
25	ESCs mtNA prep - 3	60	E3_60
27	ESCs mtNA prep - 3	90	E3_90

Samples analysed were three independent preparations of ESC mtNA, two independent preparations of liver mtNA, two independent preparations of brain mtNA and a preparation of synthetic mtDNA. Index numbers in 24 different PBAT-PE-iX-N4 (see Methods), duration of bisulfite conversion in minutes and abbreviated names are shown.

**Figure 2:**
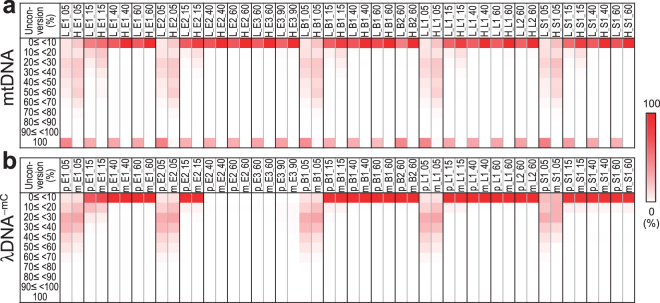
Heat map of distributions of reads acquired from next-generation bisulfite sequencing. Three independent preparations of ESC mitochondrial nucleic acid (mtNA) (E1, E2 and E3), two independent preparations of brain mtNA (B1 and B2), two independent preparations of liver mtNA (L1 and L2) and a preparation of synthetic mtDNA (S1) were subjected to bisulfite conversion for 5, 15, 40, 60 and 90 min (5, 15, 40, 60 and 90) and a total of 24 bisulfite-converted samples (Table 1) were deep-sequenced. Reads were sorted to corresponding samples with distinctions between L stands (L) and H strands (H) of mtDNA (**a**), and between plus strands (p) and minus strands (m) of spiked λDNA^−mC^ (**b**). Unconversion rates of cytosines in each read were calculated from statuses (converted or unconverted) of all cytosines in the read, and were expressed as percentages. Reads were then categorised with 10% increments of unconversion rates, and distributions of the reads in each increment block were calculated as percentages and are presented in heat maps; colour changes from white to red indicate from 0% to 100%. Calculated percentages of read distributions in increment blocks for each read assembly are shown in Supplementary Fig. S2e,f.

A curious, and probably alarming observation was that when methylation levels of individual reads were analysed, a significant fraction of reads that were mapped to L strands of mtDNA (roughly 30% of L strand-mapped reads) remained completely unconverted, in other words, no cytosine in such reads was converted, even after 1 h incubation with high-concentration bisulfite solution at 70 °C (Fig. 2a,b and Supplementary Fig. S2c–f). This phenomenon was also evident in the L strand of synthetic mtDNA, but was not seen in H strands nor either strands of λDNA^−^^mC^. Therefore, it should not be due to heavy methylation of L strands but should be attributable to certain character of the sequence of the strand. Hence, we excluded reads with ≥90% unconverted cytosine levels and proceeded to further analyses.

**Figure 3:**
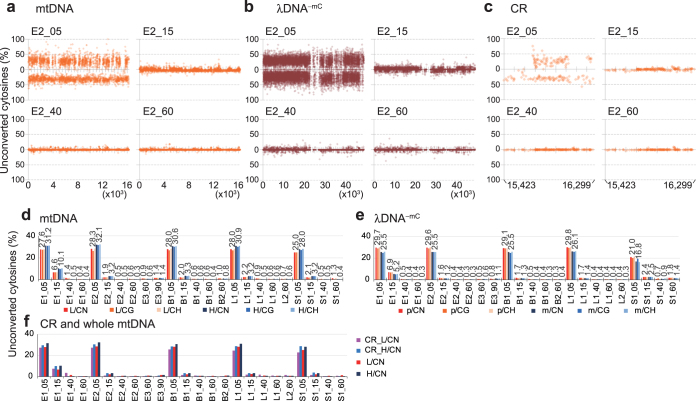
Analysis of methylation status of mtDNA by next-generation bisulfite sequencing. (**a**–**c**) Cytosine unconversion rates at cytosine sites with coverage of ≥10 in whole mtDNA of an ESC mtNA preparation (E2) (**a**), λDNA^−mC^ mixed as an internal control (**b**) and the control region (CR) of the mtDNA (**c**) are plotted according to nucleotide numbers (X axis) from 1 to 16,299 for mtDNA, from 1 to 48,502 for λDNA^−mC^ and from15,423 to 16,034 for the CR, and unconversion rates in percentages (Y axis). Nucleotide positions on X axes are shown below graph fields. Plots for L strands of mtDNA and plus strands of λDNA^−mC^ are shown above X axes with percentages of unconversion increasing upwards, and H strands and minus strands below X axes downwards. Graphs with higher resolution are shown in Supplementary Fig. S3. (**d,e**) Average unconversion rates of cytosines with coverage of ≥10 in whole mtDNA and λDNA^−mC^. For each sample shown are average unconversion rates of all cytosines in CN (N = A, G, C and T), CG and non-CG sequences in L strands and H strands of whole mtDNA (L/CN, L/CG, L/CH, H/CN, H/CG and H/CH, respectively) (**d**) and in plus strands and minus strands of λDNA^−mC^ (p/CN, p/CG, p/CH, m/CN, m/CG and m/CH, respectively) (**e**). Percentages of unconverted cytosines in L/CN, H/CN, p/CN and m/CN are shown above the bars. (**f**) Comparisons of average unconversion rates of cytosines with coverage of ≥10 in L strands and H strands of the CR of mtDNA and whole mtDNA (CR_L/CN, CR_H/CN, L/CN and H/CN, respectively). Sample names are presented as in Fig. 2.

Rates of unconverted cytosines were then calculated for cytosine sites with coverage of ≥10, and the unconversion rates of these sites in L and H strands of mtDNA and in plus and minus strands of λDNA^−^^mC^ were separately plotted along nucleotide positions (Fig. 3a–c and Supplementary Fig. S3a–f). Average unconversion levels of cytosines with ≥10 coverage were then calculated for CG, non-CG (CH) and CN sites (N = A, G, C and T) in each strand (Fig. 3d,e and Supplementary Table S2). Judging from changes in average percentages of unconverted cytosines of λDNA^−mC^ and synthetic mtDNA in bisulfite treatment time courses, 40 and 60 min were sufficient for conversion under our experimental conditions (>99% conversion in λDNA^−mC^ in all samples excepting S1_60, >98% in λDNA^−mC^ in S1_60, >99% in synthetic mtDNA in S1_40 and >98.5% in synthetic mtDNA in S1_60, in ‘CN’ columns in Fig. 3d,e). Read numbers of both mtDNA and λDNA^−mC^ decreased considerably after 90 min incubation (Supplementary Fig. S2c,d), suggesting that prolonged treatment with bisulfite caused extensive deterioration of DNA. Under these conditions, mtDNA of ESCs and tissues showed similar time-dependent transition patterns as that of synthetic mtDNA. Moreover, mean percentages of conversion at both CG and non-CG sites on L and H strands in all native mtDNA samples were >99% after 1 h bisulfite conversion, excepting those of the L strand of B2_60 mtDNA (≥98.9%) (Fig. 3d and Supplementary Table S2). Because overall observations of mtDNA did not indicate apparent methylation of mitochondrial genomes, we then examined whether mtDNA has a specific region(s) that is methylated substantially. mtDNA was divided into 500 bp non-overlapping windows with distinctions of the two strands and average levels of unconverted cytosines in each window were compared between samples after 40 and 60 min incubation with bisulfite. These comparisons with synthetic mtDNA series revealed no windows with apparently higher levels of unconverted cytosines in all native mtDNA series, and no windows had a single native mtDNA series (i.e., all ESC samples, all liver samples or all brain samples) with apparently higher unconversion rates than other native mtDNA series and synthetic mtDNA series (Supplementary Fig. S3g,h). Further, we specifically examined the control region (CR) of mtDNA, because it contains important cis-elements for replication and transcription^36^. Comparison of data from native mtDNA and synthetic mtDNA with 40 and 60 min incubation did not suggest substantial signals that were common and specific to all native samples (Fig. 3c,f, Supplementary Fig. S3c and Supplementary Table S2). Taken together, these deep-sequencing analyses suggest that 5mC is not present at any specific position(s) in mtDNA with a detectable frequency, even if mtDNA would contain it.

In addition, inspection of time-dependent changes in the patterns of bisulfite conversion of mtDNA revealed intriguing differences between strands; unconversion rates of cytosines in L strands seemed to be more variable than those in H strands (Supplementary Fig. S3a,c). These observations were supported by comparisons of standard deviations for cytosine unconversion rates between the two strands in each sample (Supplementary Fig. S3i,j). Finally, we analysed reads derived from mtDNA with ≥90% unconverted cytosine levels (Supplementary Fig. S4a). These reads were aligned to the reference mtDNA sequence and coverage numbers at cytosine sites were plotted if they are greater than 10 (Supplementary Fig. S4b,c). In these analyses, synthetic and native mtDNA gave similar patterns, suggesting that the resistance of cytosines to bisulfite conversion (Fig. 2) is not due to methylation.

### mtDNA is not affected by treatments with McrBC

**Figure 4:**
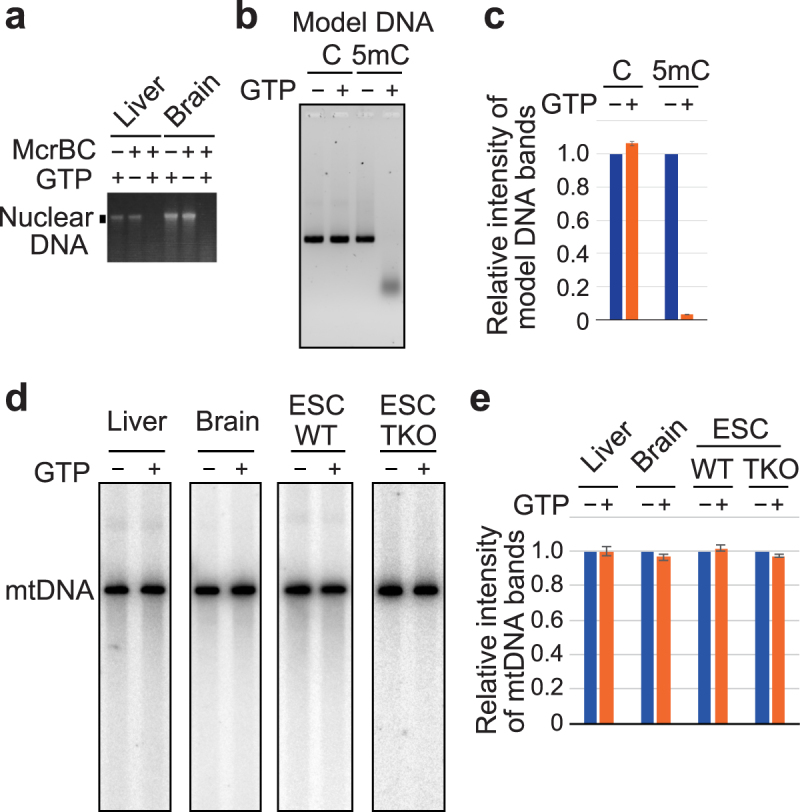
Analysis of mtDNA with methylated cytosine-sensitive endonuclease McrBC. (**a**) Ethidium bromide staining image of nuclear DNA after agarose gel electrophoresis. Samples were total nucleic acid (total NA) from mouse liver and brain tissues incubated without (−) or with (+) McrBC and without (−) or with (+) GTP. The full-length gel is presented in Supplementary Fig. S6a. (**b**) PCR-generated DNA fragments containing unmodified cytosines (C) or 5mC were treated with McrBC with or without GTP, and then were electrophoresed and visualised by Sybr Green I staining. (**c**) Quantification of the full-length band region in (**b**). Intensities of bands in −GTP lanes were expressed as 1 and the relative intensities of corresponding + GTP lanes were calculated for each experiment. Assays were performed three times and means are shown with SEM. (**d**) Southern hybridisation images of linearised mtDNA of liver, brain, wild-type ESCs (WT) and TKO ESCs (TKO) treated with McrBC in the absence or presence of GTP. Blot images with cropped regions which are necessary to be shown in this study are presented in Supplementary Fig. S6b. (**e**) Band intensities of full-length mtDNA (−GTP) were expressed as 1 and the relative intensity values of bands with McrBC cleavage (+GTP) were calculated for each experiment for each sample. Two independent preparations of samples were analysed for three times (technical triplicates) and the average of technical triplicates was given for each sample. Subsequently, means were calculated from the two average values and are presented in the graph with errors of the means.

McrBC cleaves DNA between two 5′-RmC recognition sites (R = A or G; mC = methylated cytosine), where numbers of nucleotides between the two sites are optimally 40–80 bp but can be more than 1 kbp with reduced efficiency of digestion, and cleavage is triggered by encounters of two DNA-translocating McrBCs^30,37,38^. As reported previously^39^, McrBC enzyme activity is dependent on GTP; while McrBC digested nuclear DNA of mouse tissues in the presence of GTP, the absence of GTP prevented the digestion (Fig. 4a and Supplementary Fig. S6a). Because enzyme-mediated determinations of DNA methylation could be disturbed by false positives due to non-specific DNA cleavage by possible contaminating nucleases in McrBC preparations, we controlled the activity of McrBC according to the presence of GTP. Model DNA fragments with a part of mouse mtDNA sequence were generated using PCR with dATP, dGTP, dTTP and dCTP or 5-methyl-dCTP as substrates. Although fragments with unmodified cytosines were essentially unaffected by McrBC treatments, those with modified cytosines were digested efficiently (Fig. 4b,c), confirming that McrBC cleaves DNA containing 5mC.

To examine methylation of mtDNA, we performed McrBC cleavage assays. mtNAs from ESCs and mouse tissues were initially treated with the restriction enzyme BlpI and mtDNA was linearised. Samples were then incubated with McrBC in the presence or absence of GTP, and after gel electrophoresis, mtDNAs were visualised using Southern hybridisation. As shown in Fig. 4d,e and Supplementary Fig. S6b, band intensities of the linearised mtDNAs of wild-type ESCs, liver and brain showed no substantial reduction upon treatment with McrBC. In addition, similar result was obtained when mtDNA of triple-knockout (TKO) ESCs that lack DNMT1, DNMT3A and DNMT3B was incubated with McrBC. These data suggest that mtDNAs do not contain appreciable amounts of 5mC.

### Investigation of 5mC in mtDNA by LC/MS

**Figure 5:**
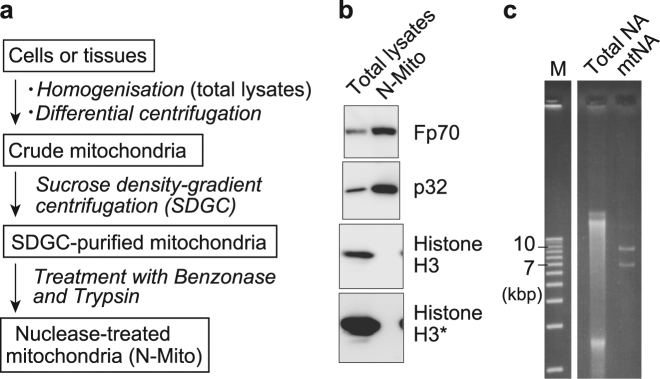
Preparation of mitochondria and highly purified mtDNA. (**a**) Flow of mitochondrial preparation with three grades of mitochondrial fractions and key experimental steps. (**b**) Western blot analysis of total cell lysates (total lysates) and nuclease/protease-treated mitochondrial preparations (N-Mito) from mouse livers using antibodies against Fp70, p32 and histone H3. The panel with histone H3* is a longer exposure image of the panel above. (**c**) An example of ethidium bromide stained image of liver total NA and liver mtNA purified from N-Mito; they were treated with the restriction enzyme EcoRV, which cuts mouse mtDNA twice to generate 9.5 and 6.8 kb fragments. While corresponding bands were not seen in total NA due to the small proportion of mtDNA compared to nuclear DNA, two bands corresponding to the EcoRV-generated mtDNA fragments were observed in mtNA from N-Mito. A DNA ladder (M) was run on the same gel but not next to the sample lanes. Full-length gel and blots are presented in Supplementary Fig. S6c,d. Note that larger amounts of mtNA were treated with EcoRV and RNase T1 and were electrophoresed for gel purification of mtDNA for LC/MS analysis.

To further investigate mtDNA methylation, we performed LC/MS analyses, which are more sensitive than the above two methods in detecting 5mC and give quantitative information. However, LC/MS analyses require pure mtDNA that is strictly free of nuclear DNA contamination, producing considerable technical challenges. Specifically, because samples are digested into mononucleosides prior to injection into an LC/MS instrument, the presence of nuclear DNA in mtDNA fractions precludes discrimination between nucleosides from mitochondrial and nuclear DNA. Thus, after preparing crude mitochondria using differential centrifugation (Fig. 5a), we further purified mitochondria using ultracentrifugation with a sucrose density gradient. Subsequently, we treated SDGC-purified mitochondrial fractions with nuclease and proteinase to degrade residual contaminating nuclear DNA and washed the mitochondria extensively to eliminate nuclear DNA (N-Mito) (Fig. 5a). The quality of mitochondrial preparations was then confirmed according to enrichment of the mitochondrial proteins Fp70 (a subunit of respiratory complex II localised in inner mitochondrial membrane), p32 (a mitochondrial matrix protein) and TFAM (a mtDNA binding protein), and successful elimination of nuclear fractions was supported by reductions in histone H3 signals in N-Mito fractions (Fig. 5b and Supplementary Figs S5 and S6c). Subsequently, mtNA was extracted from N-Mito, and mtDNA from mtNA fractions was electrophoresed, was visualised with ethidium bromide (Fig. 5c and Supplementary Fig. S6d) and was gel-purified.

**Figure 6:**
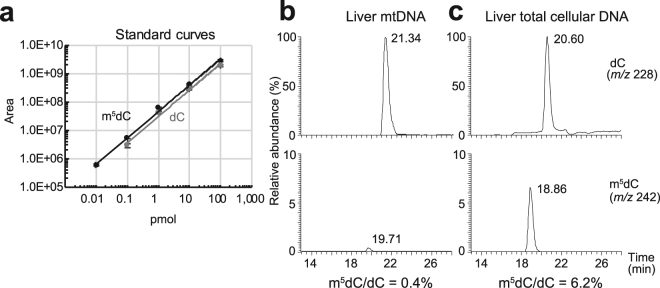
Mass spectrometric nucleoside analysis of mtDNA. (**a**) Standard curves of deoxycytidine (dC; grey line) and 5-methyldeoxycytidine (m^5^dC; black line) produced with serially diluted authentic chemicals; X and Y axes show amounts of authentic mononucleosides applied and peak areas of corresponding extracted mass chromatograms, respectively. Authentic samples were analysed three times and the means are plotted with SD. (**b,c**) Analyses of dC and m^5^dC in mtDNA (**b**) and total cellular DNA (**c**) from liver tissues. Top and bottom panels show extracted mass chromatograms for proton adducts of dC (*m/z* 228) and m^5^dC (*m/z* 242), respectively. Numbers near the peaks indicate retention times. Due to low abundance of mtDNA, results from total cellular DNA effectively represent the state of nuclear DNA. Two independent preparations were analysed once for mtDNA and twice for nuclear DNA. Mean m^5^dC/dC percentages were calculated for mtDNA and nuclear DNA.

To quantitatively compare 5-methylcytosine (5mC) and unmodified cytosine using LC/MS, standard curves of corresponding deoxyribonucleosides were generated using authentic 5-methyldeoxycytidine (m^5^dC) and deoxycytidine (dC) (Fig. 6a). The ionisation efficiency of m^5^dC was approximately 50% better than that of dC (Fig. 6a), and mouse livers were chosen to obtain sufficient quantities of mtDNA for LC/MS investigation. Taking into consideration of the data from measurements of authentic nucleosides, liver mtDNA was digested enzymatically into mononucleosides and was subjected to mass spectrometric analyses. Very weak m^5^dC signals were observed in comparison with those of dC from two independent preparations of liver mtDNA (Fig. 6b), and amounts of m^5^dC relative to dC were estimated to be 0.3% and 0.5%, which is equivalent to 18–30 5mC residues per molecule of mtDNA. Under these analytical conditions, m^5^dC was present at about 6% of dC in genomic DNA (Fig. 6c), which is consistent with previous observation^3^.

## Discussion

In this study we aimed at investigating mtDNA with unprecedented thoroughness and providing a set of solid data that enable us to make a proposition on mammalian mtDNA methylation. To this end, we employed three contrasting experimental principles to detect 5mC; namely, bisulfite sequencing, McrBC cleavage assays and LC/MS.

A crucial point of our experiment design in next-generation bisulfite sequencing was parallel analyses of PCR-generated mtDNA, which has no 5mC. Under the conditions where bisulfite conversion of λDNA^−mC^ was sufficient, about 30% of reads that were mapped to mtDNA L strands were completely unconverted. Because experiments with synthetic and native mtDNA both showed such phenomena, we excluded reads in which ≥90% of cytosines were unconverted from subsequent analyses. Even after the exclusion, a fraction of unconverted cytosines was still present in synthetic mtDNA and control λDNA, which is appreciated with the results that overall conversion rates of synthetic mtDNA and λDNA^−mC^ did not reach 100%. Considering these observations and comparing results from native mtDNA and those from synthetic mtDNA, it is reasonable to conceive that cytosines which were not converted in native mtDNA were not methylated but likely escaped from bisulfite conversion reaction as were the cases with synthetic mtDNA and λDNA^−mC^. Hence, parallel analyses of control DNAs, not only λDNA^−mC^ but also synthetic mtDNA, are critical to interpretations of data from bisulfite sequencing analyses of mtDNA. In addition, experiences in the ‘wet’ experiments could be related to the ‘dry’ analysis results that ESC mtDNA in published WGBS data gave strong signatures of L strand-specific methylation (unconversion). Collectively, the present bisulfite sequencing data suggested that mitochondria lack cytosine methylation, or that mtDNA contains 5mC in a non position-specific manner and levels of 5mC at each cytosine site are below the accredit detection sensitivity of our bisulfite sequencing methods.

Analysis of reads with unconverted cytosine levels of ≥90% after 1 h incubation with bisulfite revealed several mtDNA positions in which coverage numbers of cytosines from the reads are rather high, and these were located in the protein-coding genes (e.g. *ND2*, *ND5* and *ND6*). Because these phenomena were common to synthetic and native mtDNA, they may reflect DNA structures induced after denaturation of L and H strands of (BglII-digested) genome-length linear mtDNA, local sequences of L strands of such mtDNAs or specific experiment conditions of our study.

McrBC cleavage assays involve detection of bands of linearised full-length mtDNA with Southern hybridisation, and single cuts of linear mtDNA by McrBC will reduce band intensities. Previous studies proposed that two RmC sites that can be spaced more than 1 kb apart are required for the cleavage of DNA by McrBC^30,37,38^. In our assays, mtDNA bands were not affected by treatments of the enzyme. We also analysed mtDNA by LC/MS. Because sample preparation requires digestion of DNA into mononucleosides, LC/MS data are not supported by sequence information and trace levels of nuclear DNA contamination in mtDNA fractions would influence 5mC determinations. Therefore, we purified mtDNA from liver samples using multiple steps and 5mC nucleoside was detected from mtDNA preparations. Although 5mC levels were fairly low, they corresponded to 24 residues of 5mC per mtDNA molecule. Given that 46% of cytosines are preceded by purines (R) in mouse mtDNA, 11 5 mC residues should follow purine residues (24 residues × 0.46), and this number is equivalent to a single 5mC residue for every 1.5 kbp of mtDNA. Taken together, McrBC assays and LC/MS analyses suggest the following scenarios: [1] If most 5mCs in mtDNA are not preceded by purines, the number of 5mCs that can be used for cleavage of mtDNA by McrBC is considerably less than 11 per molecule of mtDNA, and thus it might have been difficult to observe reductions of mtDNA bands in cleavage assays. In this case, 5mC detected in LC/MS investigation would be derived genuinely from mouse liver mtDNA. A possibility could then be that DNMT1, DNMT3A or DNMT3B is imported into mitochondria to methylate cytosine residues. However, mitochondrial localisation of DNMTs remains controversial^29^. [2] LC/MS detection of 5mC from liver mtDNA may entirely or partly reflect the presence of trace contaminations with nuclear DNA in mtDNA preparations, despite our extensive purification procedures. Because the mass of mtDNA is less than 1% of that of nuclear DNA, it would be difficult to completely remove nuclear DNA from mtDNA preparations, and 5mC levels in liver mtDNA from LC/MS analyses (0.3–0.5%) would be an overestimation. This idea seems to fit with the results that bisulfite sequencing did not reveal any location where mtDNA methylation occurs and that mtDNA band intensities were not significantly reduced after McrBC treatments. [3] It is possible that both of these scenarios contribute to the present data. In any scenario, with the conclusions of our bisulfite sequencing analyses, such low levels of 5mC (at the most 0.3–0.5% of unmodified cytosine) are unlikely to play a role in mtDNA gene expression or mitochondrial metabolism.

Hong *et al*.^26^ studied methylation of mtDNA in human cells using bisulfite sequencing as the sole method of their analyses and proposed that cytosine methylation is effectively absent in mtDNA. Herein, we investigated mtDNA of mouse ESCs, liver and brain using bisulfite sequencing, McrBC digestion assays and LC/MS, and reached our conclusions. Furthermore, our careful application of bisulfite sequencing to mtDNA indicated difficulty in obtaining true/accurate information on mtDNA methylation with this method. Appropriate controls are essential for monitoring of bisulfite conversions and the ensuring data analyses. These were internal control DNA that was spiked into sample solutions (λDNA^−mC^ in this study) and an authentic methylation-free DNA that carries the mtDNA sequence (synthetic mtDNA). In the absence of these controls, the validity of bisulfite sequencing results should be interrogated carefully, particularly because failures of conversion of unmodified cytosines are considered indicative of methylation. Mechta *et al*.^40^ also performed bisulfite sequencing using a next-generation sequencer and discussed concern about mtDNA methylation analysis with bisulfite sequencing methods.

A recent bisulfite sequencing study of the CR of mtDNA reported 100% methylation of numerous cytosines at CG and non-CG sites of L strands^21^. The absence of unconverted cytosines in PCR products with mtDNA sequences was considered as the ground for efficient bisulfite conversion of native mtDNA. We suggest that because their control PCR products were only a few hundred bp long, short fragments with mtDNA sequences were bisulfite-converted, whereas L strands (of the CR) of native mtDNA, which is 16.3 kbp, may not have been converted efficiently. Another recent study^24^ also examined the CR and the cytochrome *c* oxidase subunit I coding region of mtDNA using bisulfite sequencing, and suggested that cytosines at both CG and non-CG sites in H and L strands were strongly methylated, with average methylation levels of ca. 20%–40%. However, because it appears that the study neither had spiked control DNA in sample solutions nor performed parallel experiments with control DNA having the corresponding mtDNA sequence, whether or not mtDNA was completely bisulfite-converted was not adequately examined. Infantino *et al*.^15^ proposed that 25% of cytosines in mtDNA were modified into 5mCs from mass spectrometric analysis. This is a rather high level, as even in nuclear DNA only several percent of cytosines is methylated. Also, if 5mC residues were present at such a high frequency, all of the present experiments should have detected substantial methylation. Shock *et al*.^16^ performed immunoprecipitation of sheared mtDNA using anti-5mC antibodies and suggested the presence of 5mC in mtDNA with qPCR amplification of precipitated fragments, although one cannot know the actual levels of methylation they detected from their description. Moreover, because their immunoprecipitation was not combined with bisulfite sequencing analyses^16^, it is possible that their observations were stemmed from weak non-specific binding of antibodies to unmodified cytosines when the sample DNA contains no or extremely few 5mC.

In summary, our data suggest that 5mC is not present at any specific position(s) in mtDNA and that levels of the methylated cytosines are very low, provided the modification occurs. In addition, we revealed alarming aspects of bisulfite sequencing upon applying to mtDNA. We infer that these features make investigations of mtDNA unexpectedly complex and thus have produced current controversies regarding the methylation of mtDNA^29^, despite the considerable research efforts of the past 4 decades^7–26,40–43^. Although it remains possible that mtDNA is methylated to the extent of nuclear DNA under as yet uncharacterised conditions, we provide accurate estimation of 5mC in mammalian mtDNA and hope that our data resolve current controversies among studies of mtDNA methylation.

## Methods

### Analysis of published WGBS data

WGBS data were obtained from public depositories and nucleotides with phred scores below 20 were trimmed from WGBS reads using Perl script. The resulting reads were aligned to reference genome sequences of mouse (mm10) or human (hg19) and reference mtDNA sequences of mouse (NC_005089.1) or human (NC_001807.4) using Bismark alignment software (version 0.14.0)^35^. Several WGBS datasets used in this study were produced from DNA libraries that were generated using the PBAT method^34^. Alignments of these WGBS data were performed in Bismark using the option “--pbat”. Reads that were not mapped to nuclear DNA but were to mtDNA reference sequences were then extracted. Subsequently, reads whose averaged methylation levels of all cytosines in non-CG sequence (non-CG methylation levels) were ≥0.7 as well as whose strand contained an G to A substitution were discarded. The retained reads were used to calculate methylation/unconversion levels of each cytosine site of mtDNA with coverage (read depth) of ≥10 using the following formula: the number of methylated reads / the number of methylated and unmethylated reads, and then average methylated/unconverted cytosine levels in mtDNA were obtained. Methylation levels of nuclear DNA were calculated using reads that were aligned to nuclear DNA reference sequences using the same conditions for mtDNA analysis.

### Cell culture and mice

Wild-type ESCs (E14) were cultured on feeder cells in 2i-LIF medium consisting of Neurobasal Medium (Life Technologies), DMEM/Ham’s F-12 medium (Wako Pure Chemical Industries), 1.9 mM glutamine (Nacalai tesque), N-2 (GIBCO), B-27 (GIBCO), penicillin-streptmycin, leukemia inhibitory factor (LIF) and two inhibitors (2i); GSK-3β inhibitor CHIR99021 and MEK inhibitor PD0325901. Medium was changed daily and cells were subcultured by trypsinisation every 2–3 days. Feeder cells were generated using X-ray irradiation of mouse STO fibroblast cells. TKO ESCs lacking in DNMT1, DNMT3A and DNMT3B (*Dnmt1*-/- *Dnmt3a*-/- *Dnmt3b*-/-)^44^ were cultured on Matrigel matrix (Corning) in the same medium described above. To avoid contamination of TKO ESC preparations with cells expressing DNMTs, feeder cells were not used for TKO ESC cultures. E14 ESCs, TKO ESCs and STO cells were kind gifts from Dr. H. Koseki (RIKEN), Dr. M. Okano (Kumamoto Univ.) and Prof. K. Hayashi (Kyushu Univ.), respectively. Liver and brain samples were obtained from wild-type C57BL/6 J mice in accordance with the guidelines of and with approval of the animal ethics committee of Kyushu University Graduate School of Medicine, Japan and liver samples from wild-type ICR mice were purchased from Kyudo Co. Ltd. (Saga, Japan).

### Preparation of mitochondria

Mitochondria were prepared as described previously^45^ with modifications. Typically, ESCs were harvested at room temperature from 25 9 cm culture dishes for a single preparation, or fresh mouse liver and brain tissues were minced well using scissors in a cold room. Where appropriate, the following procedures were performed at 4 °C or on ice. Samples were then homogenised in H buffer (225 mM mannitol, 75 mM sucrose, 10 mM Hepes-NaOH, 2 mM EDTA and 2-mercaptoethanol) using a Teflon homogeniser. Homogenates were centrifuged at 900/600 g (ESCs/tissue) to precipitate nuclei and undisrupted cells (P1). Supernatants were then centrifuged and the resulting supernatants were collected (S1). P1 pellets were resuspended in H buffer and were centrifuged at 900/600 g (ESCs/tissue) and supernatants were collected (S2). S1 and S2 supernatants were then mixed and centrifuged at 12,000/5,000 g (ESCs/tissue) and precipitates were resuspended in H buffer and centrifuged again. This step was repeated once for tissue samples, and crude mitochondria were obtained as precipitates, were resuspended in a small volume of H buffer and were then subjected to sucrose density step gradient (1 M/1.5 M) centrifugation at 15,700 rpm using a SW41 Ti rotor (Beckman Coulter). Purified mitochondria between the two layers of sucrose solution were recovered and centrifuged at 12,000 g and the resulting sucrose density gradient centrifugation (SDGC)-purified mitochondrial pellets were resuspended in E buffer (225 mM mannitol, 75 mM sucrose, 10 mM Hepes-NaOH) with 1 M KCl, and were then centrifuged again. To eliminate nuclear contaminants, 5 mg/ml mitochondria in E buffer with 2 mM MgCl_2_ were incubated with 50 units/ml Benzonase (Merck) at 30 °C for 15 min, followed by addition of 2.5 µg/ml trypsin and further incubation for 15 min. Enzyme reactions were terminated by adding equal volumes of stop buffer (E buffer with 25 mM EDTA and a proteinase inhibitor cocktail), and the mitochondria were then recovered as precipitates by centrifugation. Mitochondrial pellets were then resuspended and centrifuged three times in fresh aliquots of stop buffer to produce ‘nuclease-treated’ mitochondria (N-Mito).

### Western blot analysis

Protein lysates prepared from whole cells or mitochondria were electrophoresed on SDS-polyacrylamide gels and were blotted onto PVDF membranes (Millipore). Proteins were detected using the following primary antibodies: anti-histone H3 (Cell Signaling Technology #4499), anti-Fp70 (ThrmoFisher Scientific #459200), anti-p32/gC1qR^46^ and anti-TFAM^46^. Secondary antibodies used were HRP conjugates. Protein bands were detected using commercial detection kits and an ImageQuant LAS4000 instrument (GE Healthcare).

### Preparation of mtNA

N-Mito prepared from ESCs and tissues were lysed with Lysis buffer [75 mM NaCl, 50 mM EDTA (pH 8), 10 mM Hepes-NaOH (pH 7.2), 1% SDS and 0.2 mg/ml Proteinase K] and lysates were incubated at 50 °C for 30 min. mtNA was then extracted sequentially with phenol and chloroform/isoamyl-alcohol (CI) and was precipitated with 2-propanol. mtNA pellets were then rinsed in 70% ethanol, and were air-dried and dissolved in 10 mM Hepes-NaOH (pH 7.2) or H_2_O. (The procedures of rinse and air-dry of pellets were followed where alcohol precipitation of nucleic acids was performed in sections below.)

### Bisulfite sequencing of mtDNA by cycle sequencing

Three independent preparations of mtNA from N-Mito of E14 ESCs (ESC preps. i−iii in Fig. 1) were incubated with the restriction enzyme DraI to cleave mtDNA at several sites (Supplementary Fig. S2a). Samples were then subjected to sequential extraction using phenol and CI and ethanol precipitation. Bisulfite conversion was performed as previously described^47,48^ with modifications. Fifty ng of mtNA samples containing DraI-digested mtDNA were mixed with 100 ng of unmethylated lambda DNA (λDNA^−mC^) (Promega) and were then denatured by heating at 90 °C for 3 min in 10 mM Hepes-NaOH (pH 7.2) and 2 mM EDTA (pH 8) in 11.25 µl. Subsequently, 137.5 µl aliquots of 10 M bisulfite solution mixed with 1.25 µl aliquots of 3 N NaOH were added to the denatured samples, and bisulfite conversion reactions were performed at 70 °C for 1 h. (Our original protocol^47,48^ denatures DNA using NaOH. Thus, NaOH solution was added to bisulfite solution prior to conversion reactions to faithfully apply the conditions of bisulfite conversion in the original protocol.) The resulting nucleic acid samples were further processed to complete the bisulfite conversion reactions using EZ DNA methylation kit (Zymo Research); the samples were applied to columns, were treated with M-desulphonation buffer and were eluted with H_2_O.

A part of the cyt*b* gene from bisulfite-treated mtDNA was strand-specifically amplified using TaKaRa Ex Taq DNA polymerase (TaKaRa) with the following PCR primer pairs (Y = C and T; R = A and G): 5′-GCACACTGAATTTGAGGGGGYTTYTYAGTAGAYA-3′ and 5′-CAGCACCACCACATTACTARRATTCARTACAAAATTTRTRTRATT-3′, and 5′-AAGGAAAGGTATTAGGGYTAAAATT-3′ and 5′-ACCTCCTATCARCCATCCCATATAT-3′, which amplified nucleotides (nt) 14,632–15,130 of the L strand and 15,065–14,587 of the H strand in the cyt*b* gene, respectively. Underlined nucleotides are tails for second PCR (see below) and do not correspond to mtDNA sequences. Because cytosine positions in mtDNA could be occupied by either modified or unmodified cytosines, nucleotides in the PCR primers corresponding to C in mtDNA were randomised with Y in the sense primers to the corresponding strands and with R in the anti-sense primers so that the primers could hybridise to the target regions of the specified strands and the PCR-synthesised complementary strands with all possible patterns of cytosine methylation. A region in the converted λDNA^−mC^ was amplified using the primer pair of 5′-GGGTGATTTTTATTAAAGGGGTATT-3′ and 5′-TACACCATCCTCTTCCTACAAACTC-3′^49^. Primers were designed to amplify the region from nt 2,164 to 2,576 (GenBank accession: NC_001416.1) of bisulfite-converted λDNA^−mC^. PCR was conducted with 20 cycles of 95 °C for 30 s, 60 °C–50.5 °C (−0.5 °C/cycle) for 30 s and 72 °C for 2 min (touch-down PCR), followed by 15 cycles of 95 °C for 30 s, 50 °C for 30 s and 72 °C for 30 s and the final extension at 72 °C for 10 min^49^. PCR-amplified DNA fragments from H strands and λDNA^−mC^ were gel-purified. In the case of L strand, PCR-amplified fragments were purified using AmpureXP (Beckman Coulter) and second PCR was performed using the purified fragments as templates and the primer pair 5′- GCACACTGAATTTGAGGG-3′ and 5′-CAGCACCACCACATTAC-3′ with 4 cycles of 98 °C for 10 s, 54 °C for 30 s and 72 °C for 1 min, and the resulting fragments were then gel-purified. Gel-purified fragments derived from H strands, L strands and λDNA^−mC^ were ligated into pGEM-T Easy vectors (Promega) and plasmids were transformed into competent *E. coli* cells. Colonies were then picked up and inserted fragments were examined. Those with expected lengths after gel electrophoresis were sequenced using cycle sequencing. Insert sequences were then aligned with reference sequences using ATGC Sequence Assembly Software (GENETYX Inc., Japan) and the methylation status of mtDNA was analysed using the QUMA programme^50^.

### Bisulfite sequencing of mtDNA using next-generation sequencing

mtNAs from E14 ESCs and liver and brain tissues were treated with the restriction enzyme BglII to linearise mtDNA with a single cut at nt 15,338, followed by sequential extraction using phenol and CI and ethanol precipitation. Synthetic mtDNA mimicking the BglII-digested native mtDNA (Supplementary Fig. S2a) was generated using PCR with standard dNTP and the primers 5′-CTCTTCTCAAGACATCAAGAAGAAGGAGC-3′ and 5′-CATTTCAGGTTTACAAGACCAGAGTAATGTTTATACTATC-3′. PCR products were gel-purified and the resulting synthetic mtDNA was 16,291 bp long spanning from nt 15,342 to 15,333 and was free from 5mC.

Three independent preparations of ESC mtNA, two independent preparations of liver mtNA, two independent preparations of brain mtNA (all treated with BglII) and a preparation of synthetic mtDNA were spiked with λDNA^−mC^ and were then subjected to the bisulfite conversion steps as described above with the indicated duration of bisulfite conversion (Table 1). Twenty-four samples were individually bisulfite-converted (Table 1) and were then subjected to library production using the PBAT method^34,51^. For each sample, first strand synthesis was performed using Klenow Fragment (3′ → 5′ exo-) (New England Biolabs) with Bio-PEA2-N4 (5′-biotin-ACACTCTTTCCCTACACGACGCTCTTCCGATCTNNNN -3′). Biotinylated fragments were then captured using Dynabeads M-280 Streptavidin (Life Technologies) and second strands were synthesised from captured fragments using Klenow Fragment (3′ → 5′ exo-) with PBAT-PE-iX-N4 (5′-CAAGCAGAAGACGGCATACGAGATXXXXXXGTAAAACGACGGCCAGCAGGAAACAGCTATGACNNNN-3′). Twenty-four different PBAT-PE-iX-N4 containing specific six-nucleotide index sequences (XXXXXX)^51^ were used to be compatible with subsequent multiplex sequencing. After removal of the first strand, the second strand was double-stranded using Phusion Hot Start II DNA Polymerase (Thermo Fisher Scientific) with Primer-3 (5′-AATGATACGGCGACCACCGAGATCTACACTCTTTCCCTACACGACGCTCTTCCGATCT-3′). DNA concentrations of the generated libraries were estimated using a KAPA Library Quantification kit (KAPA Biosystems). Because the concentrations of these libraries were less than 2 nM, as required for sequencing, the libraries were amplified modestly using PCR as follows. To normalise the effects of PCR on individual libraries, DNA concentrations were adjusted to the lowest of the 24 libraries and library amplification was performed with 15 cycles of PCR using the primer pair 5′-AATGATACGGCGACCACCGA-3′ and 5′-CAAGCAGAAGACGGCATACGA-3′. After amplification, all libraries were >2 nM and ran on an agarose gel as smears of 300–650 bp, confirming proper size distributions of DNA fragments in the libraries (Supplementary Fig. S2b). Finally, all amplified PBAT libraries were adjusted to 2 nM and were mixed in equal volumes. The mixture was then processed as described previously^34^ and was subjected to 50-bp paired-end sequencing on a MiSeq (Illumina) with PhiX Control kit v3 (Illumina). First reads were used for mapping and methylation analyses. After adaptor sequences and low-quality nucleotides were removed using TrimGalore (http://www.bioinformatics.babraham.ac.uk/projects/trim_galore/), the reads were mapped to the mouse mtDNA (chrM, mm10) using Bismark^35^ with default parameters and the “--pbat” option. Output data (bismark_bt2.txt files) were further processed using Perl scripts to analyse methylation (unconversion) states of cytosine residues.

### Preparation of model DNA fragments with or without 5mC

Model DNA fragments with a part of mouse mtDNA sequence from nt 16,033 to 15,510 were generated using PCR with dATP, dGTP, dTTP and dCTP or 5-methyl-dCTP (Zymo Research), TaKaRa EX Taq (TaKaRa) and the primers 5′-ATCAATGGTTCAGGTCATAAAATAATCATCAAC-3′ and 5′-GCCTTAGGTGATTGGGTTTTGC-3′. PCR products were then purified from agarose gels using QIAquick Gel Extraction Kit (Qiagen), were extracted sequentially with phenol and CI and were alcohol-precipitated.

### McrBC treatments and Southern hybridisation

mtNA was treated with the restriction enzyme BlpI, which cleaves mouse mtDNA once, followed by treatment with Proteinase K, extraction with phenol and CI and alcohol precipitation. Samples were then incubated with 10 units of McrBC (New England Biolabs) in the buffer provided by the company in the presence or absence of 1 mM GTP in 20 µl reaction volumes at 37 °C for 4 h. Reaction mixtures were then treated with Proteinase K and were electrophoresed in agarose gels, and the gels were processed for Southern hybridisation and blotted onto Zeta-Probe Membranes (Bio-Rad Laboratories). After fixation of blotted DNA using UV cross-linking, Southern hybridisation was performed using radiolabelled probes as described previously^45^ with minor modifications. Membranes were then exposed to phosphorimaging plates and bands were visualised and quantified using a Typhoon FLA9500 instrument (GE Healthcare). DNA probes were radiolabeled using Random Primer DNA Labeling Kit Ver.2 (TaKaRa) and [α-^32^P] dCTP (3000 Ci/mmol) (PerkinElmer) using PCR fragments of mouse mtDNA spanning nt 16,033–15,510.

### Purification of mtDNA and LC/MS analysis

Purified mtNA from N-Mito was treated with the restriction enzyme EcoRV to generate two mtDNA fragments of 9.5 kb and 6.8 kb. RNase T1 was added to the reaction mixture to degrade mitochondrial RNA. The reaction was terminated by sequential extraction of nucleic acids using phenol and CI, and mtDNA was precipitated with 2-propanol. Samples were then electrophoresed in agarose gels and mtDNA was visualised using ethidium bromide. Gels containing mtDNA were then excised and mtDNA was extracted using QIAquick Gel Extraction Kit (Qiagen) according to the manufacturer’s instructions, and was then ethanol precipitated.

LC/MS analysis of nucleosides was performed as described previously^52^ with some modifications. Purified mtDNA was enzymatically digested into mononucleosides as follows. mtDNA was first treated with 0.1 units of nuclease P1 (Wako Pure Chemical Industries) in 50 mM ammonium acetate (pH 7.3) at 37 °C for 1 h. Subsequently, 0.17 units of phosphodiesterase I (Worthington Biochemical Corporation) and 50 mM ammonium bicarbonate were added to reaction mixtures, followed by 1 h incubation at 37 °C. Finally, 0.1 units of bacterial alkaline phosphatase (*E.coli* C75) (TaKaRa) were added to reaction mixtures and they were incubated at 37 °C for 1 h. The resulting digests were then evaporated, were dissolved into 90% acetonitrile/ 10% H_2_O and were applied to a Q Exactive mass spectrometer (Thermo Fisher Scientific) equipped with an ESI source and an Ultimate 3000 liquid chromatography system (Thermo Fisher Scientific). Nucleosides were separated using a ZIC-cHILIC column (2.1 mm × 150 mm, Merck Millipore) with 5 mM ammonium acetate (pH 5.3) (solvent A) and acetonitrile (solvent B) at a flow rate of 0.1 ml/min in a linear gradient of 90–50% B from 0 to 30 min. Serial dilutions of authentic deoxycytidine (dC) (Wako Pure Chemical Industries) and 5-methyldeoxycytidine (m^5^dC) (Tokyo Chemical Industry) were used to generate standard curves and relative ionisation efficiencies of nucleosides were calculated. The quantities of dC and m^5^dC from mtDNA samples were then estimated using these standard curves. In addition, we analysed nucleoside contents of the nuclear DNA by subjecting total cellular nucleic acid (total NA) to LC/MS analyses.

### Data Availability

Data that support this study are deposited in Gene Expression Omnibus under the accession number GSE104104.

## Electronic supplementary material

Supplementary Information
