# Supplementary material for: Accurate estimation of 5-methylcytosine in mammalian mitochondrial DNA
Source: Sci Rep. 2018 Apr 11;8:5801. doi: 10.1038/s41598-018-24251-z (PMC5895755; doi:10.1038/s41598-018-24251-z)
Supplement: Supplementary file 1 — Supplementary Information [file 41598_2018_24251_MOESM1_ESM.pdf]

Accurate estimation of 5-methylcytosine in mammalian mitochondrial DNA

Shigeru Matsuda, Takehiro Yasukawa, Yuriko Sakaguchi, Kenji Ichiyanagi, Motoko Unoki, Kazuhito Gotoh, Kei Fukuda, Hiroyuki Sasaki, Tsutomu Suzuki and Dongchon Kang

* SUPPLEMENTARY INFORMATION *

Legends for Supplementary Figures

Supplementary Figure S1. Analysis of mtDNA reads in published whole genome bisulfite sequencing (WGBS) datasets

From published WGBS metadata, reads that were derived from mtDNA were extracted and aligned as described in the Methods section. Average cytosine unconversion rates in CG, CHG and CHH sequences (H = A, G and T) of mtDNA L strands (**a**) and H strands (**b**) of indicated mouse samples are shown as bar graphs. WGBS datasets used were SRX209455 for liver, SRX209452 for heart, SRX209454 for kidney, SRX209451 for cortex, SRX209449 for cerebellum, SRX210604, SRX210603 and SRX210602 for ESC (2i), SRX210599, SRX210600 and SRX210601 for ESC_1 and GSE30202 for ESC_2. According to literature (see References for Supplementary Table S1), ESC (2i) were cultured in 2i-LIF medium and were maintained in the ground state, and ESC_1 and ESC_2 were cultured in Serum-LIF medium and were maintained in the primed state. Superscript numbers in parentheses at shoulders of sample names correspond to those shown on the first line of Supplementary Table S1.

Supplementary Figure S2. Data analysis of next-generation bisulfite sequencing of mtDNA

(**a**) Schematic drawing of mouse mtDNA with key information for this study. The black circle represents mouse mtDNA (16,299 bp). Black arrows point to the positions of indicated mtDNA nucleotide numbers. The thick cyan line shows the control region (CR). Black and grey arrowheads

indicate restriction sites of BglII (1 site) and DraI (9 sites), respectively. The magenta line outside the mtDNA black circle indicates the position that was analysed using cycle sequencing-based bisulfite sequencing (Figure 1). The yellow line inside the black circle indicates the linear fragment of synthetic mtDNA (16,291 bp) used as the methylated cytosine-free mtDNA mimicking BglII-digested mtDNA.

(**b**) Confirmation of DNA libraries for Illumina Miseq analysis. Samples that were subjected to library generation were three independent preparations of ESC mtNA (E1, E2 and E3), two independent preparations of liver mtNA (L1 and L2), two independent preparations of brain mtNA (B1 and B2) and a preparation of synthetic mtDNA (S1). Bisulfite conversions were performed for 5, 15, 40, 60 and 90 min (5, 15, 40, 60 and 90). Fractions from the 24 libraries were individually electrophoresed in an agarose gel and were stained with Sybr Green I (top). Yellow dotted lines on the image indicate 300 bp and 650 bp positions. M indicates DNA size markers. Concentrations (DNA conc.) of the libraries are shown as a bar graph (bottom). The numbers above the gel image and below the X axis of the graph correspond to index numbers of PBAT-PE-iX-N4 (see Table 1 and Methods in the main text).

(**c,d**) Numbers of uniquely mapped reads (DNA fragments) obtained from Miseq analysis. Reads were sorted to corresponding samples according to index sequences and to the strands from which they were derived; (**c**) mtDNA L strands (L) and H strands (H) and (**d**) λDNA^{-mC} plus strands (p) and minus strands (m). Grey bars indicate numbers of all reads sorted to each category and black bars indicate numbers of reads with cytosine unconversion rates of < 90% (i.e., if a read contains 10 cytosines and 9 or all of them were unconverted, it was included in a grey bar but not in a black bar). Only numbers of reads from mtDNA L strands showed substantial differences between black and grey bars. A possible reason for the phenomenon is explained in the main text (Page 6, bottom line and Page 7, first line). Another possibility could be that if 'second round' first strand synthesis fortuitously occurred on first strand synthesis products that were synthesised with H strand fragments during the first strand synthesis step of library production, reads generated from the second round product would be considered to have DNA sequences complementary to the bisulfite-converted H strand. If the H strand fragment contained no cytosine, reads from the second round product will have no G:A mismatch against the reference L strand and would hence be aligned to the L strand. G in such a H strand fragment will be considered as an unconverted cytosine in the reads generated from the second round product.

Two hundred ng of either mtNA or synthetic mtDNA was mixed with 2 ng λDNA^{-mC} for deep sequencing. Hence, the relative ratio of synthetic mtDNA to λDNA^{-mC} was significantly higher than that of native mtDNA to λDNA^{-mC}, as mtNA contains mtDNA and mitochondrial RNAs (rRNA,

tRNA and mRNA). Because proportions of numbers of reads reflect ratios of mtDNA and λDNA^{-mC} contents, it was reasonable that read numbers of λDNA^{-mC} in synthetic mtDNA samples were low.

(**e,f**) Heat map presentation of reads acquired from deep sequencing. Refer to the legend for Figure 2 for details.

Supplementary Figure S3. Data analysis of next-generation bisulfite sequencing after exclusion of reads with cytosine unconversion rates of ≥ 90%

(**a–c**) Rates of cytosine unconversion at cytosine sites where coverage was ≥ 10 in whole mtDNA (**a**), λDNA^{-mC} mixed as an internal control (**b**) and the CR of mtDNA (nucleotides 15,423–16,034) (**c**) were plotted according to nucleotide numbers (X axis) and percentage unconversion rates (Y axis). Plots for L strands of mtDNA and plus strands of λDNA^{-mC} are shown above X axes with percentage unconversion rates increasing upwards, and H strands and minus strands below X axes with percentage unconversion rates increasing downwards.

(**d–f**) Numbers of cytosine sites with ≥ 10 coverage; mtDNA (**d**), λDNA^{-mC} (**e**) and the CR (**f**). Numbers from L (or plus) strands and H (or minus) strands are shown separately as orange and blue bars for each sample. These data were produced after exclusion of reads with cytosine unconversion rates of ≥ 90%. Numbers of total cytosine sites in the strands are shown (total number) at the right end of the graphs.

(**g,h**) From nucleotide (nt) 1 to 16,299, mtDNA was divided into 32, 500 bp non-overlapping windows and a 299 bp window (window [1]; nt 1–500, window [2]; nt 501–1,000, …, window [32]; nt 15,501–16,000 and window [33]; nt 16,001–16,299). Analysed were native and synthetic mtDNA that were subjected to 40 and 60 min incubation with bisulfite. Means of cytosine unconversion rates at cytosine sites with coverage of ≥ 10 were calculated with distinctions of L strands (**g**) and H strands (**h**) for each sample in each window, and means are shown in bar graphs. Where no bar is shown, the averaged unconversion rate was 0%. Where bars are shown below the baseline, unconversion rates in windows of the corresponding samples were not obtained because no cytosine in the sample in the window gave unconversion rate.

(**i, j**) Comparison of standard deviations (SD) of cytosine unconversion rates in L and H strands of whole mtDNA (**i**) and the CR (**j**). Average cytosine unconversion rates are presented in Figure 3d,f. Orange and blue bars show SD of cytosine unconversion rates in L and H strands, respectively. L strand showed larger deviations in all samples. Identity of samples is shown as abbreviation as in Supplementary Figure S2.

Supplementary Figure S4. Analysis of reads that were resistant to bisulfite conversion in next-generation bisulfite sequencing

(**a**) Numbers of reads from mtDNA with cytosine unconversion rates of ≥ 90%. Reads were sorted to corresponding samples according to index sequences.

(**b,c**) Distribution of cytosines in the reads with cytosine unconversion rates of ≥ 90%. Cytosine sites with coverage of > 10 were plotted. X axis, nucleotide position; Y axis, coverage number (upwards, L strand; downwards, H strand). Nucleotide positions and a gene map of mouse mtDNA are shown as the linearised form in (**b**). Synthetic and native mtDNA gave similar patterns. Identity of samples is shown as abbreviation as in Supplementary Figure S2.

Supplementary Figure S5. Western blot analysis of purified mitochondria

Western blot analyses of total cell lysates (total lysates) and nuclease/protease-treated mitochondrial preparations (N-Mito) from mouse tissues and ESCs were performed using antibodies against Fp70 (a subunit of respiratory complex II localised in inner mitochondrial membrane), p32 (a mitochondrial matrix protein), TFAM (a mtDNA binding protein) and histone H3 (a nuclear DNA binding protein). Panels with histone H3* are intensified or longer exposure images of the corresponding panels above. For wild-type ESCs, SDGC-purified mitochondrial fractions (SDGC-Mito) were also analysed. Data from liver samples presented in Figure 5 are shown for comparison. Full-length blots are shown in Supplementary Fig. S6c.

Supplementary Figure S6.

(**a**) EtBr-staining gel image corresponding to Fig. 4a. Major bands observed at the lower part of the gel (*) are most likely to be cytoplasmic ribosomal RNAs (rRNAs) and transfer RNAs (tRNAs). M indicates DNA size markers.

(**b**) Southern hybridisation images corresponding to Fig. 4d. Migration positions of mtDNA in gels varies between the blots.

(**c**) Western blot images corresponding to Fig. 5b and Supplementary Fig. S5. Numbers at the right or left sides of the panels indicate approximate positions of protein size markers. Panels of brain samples are not accompanied by size markers because they were run on the same gels as liver samples.

(**d**) EtBr-staining gel images corresponding to Fig. 5c. Major bands observed at the lower part of the gel (*) are most likely to be cytoplasmic rRNAs in the total NA lane and mitochondrial rRNAs and tRNAs in the mtNA lane. M indicates DNA size markers.

References for Supplementary Table S1

1 Hon, G. C. *et al.* Epigenetic memory at embryonic enhancers identified in DNA methylation maps from adult mouse tissues. *Nature Genet.* **45**, 1198-1206 (2013).

2 Ficz, G. *et al.* FGF signaling inhibition in ESCs drives rapid genome-wide demethylation to the epigenetic ground state of pluripotency. *Cell Stem Cell* **13**, 351-359 (2013).

3 Stadler, M. B. *et al.* DNA-binding factors shape the mouse methylome at distal regulatory regions. *Nature* **480**, 490-495 (2011).

4 Raddatz, G. *et al.* Dnmt2-dependent methylomes lack defined DNA methylation patterns. *Proc. Natl. Acad. Sci. U.S.A.* **110**, 8627-8631 (2013).

5 Shirane, K. *et al.* Mouse oocyte methylomes at base resolution reveal genome-wide accumulation of non-CpG methylation and role of DNA methyltransferases. *PLoS Genet.* **9**, e1003439; 10.1371/journal.pgen.1003439 (2013).

6 Wang, L. *et al.* Programming and inheritance of parental DNA methylomes in mammals. *Cell* **157**, 979-991 (2014).

7 Kobayashi, H. *et al.* High-resolution DNA methylome analysis of primordial germ cells identifies gender-specific reprogramming in mice. *Genome Res.* **23**, 616-627 (2013).

8 UCSD Human Reference Epigenome Mapping Project (GSE16256).

9 Lister, R. *et al.* Human DNA methylomes at base resolution show widespread epigenomic differences. *Nature* **462**, 315-322 (2009).

10 Laurent, L. *et al.* Dynamic changes in the human methylome during differentiation. *Genome Res.* **20**, 320-331 (2010).

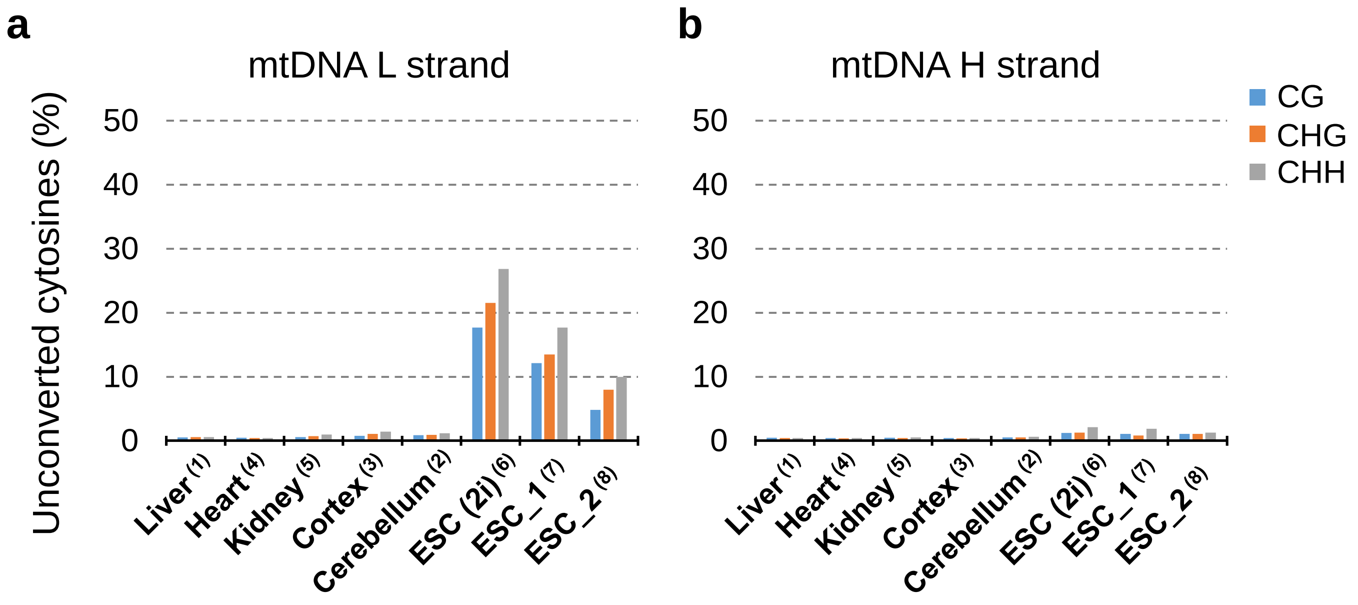

Supplementary Figure S1

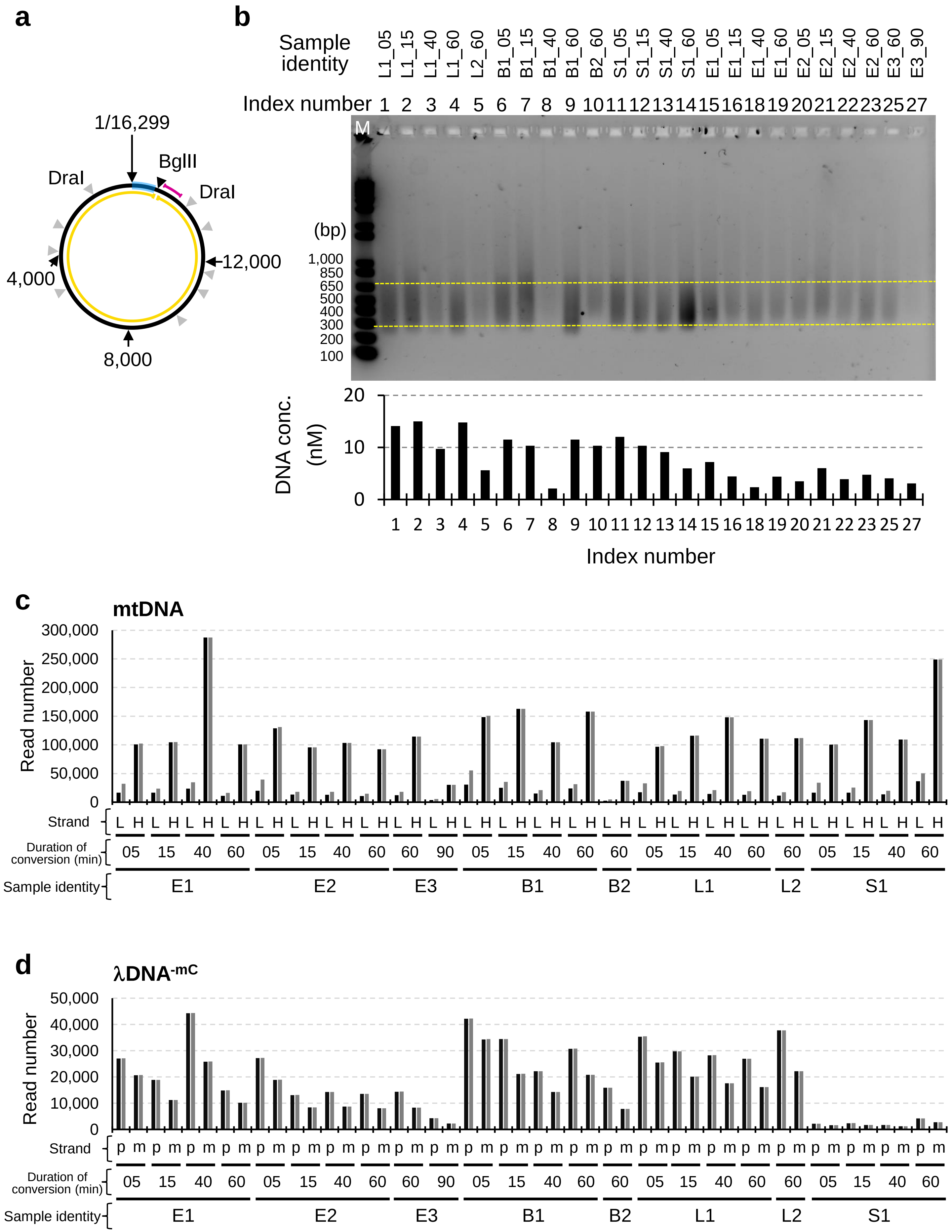

Supplementary Figure S2

e **mtDNA**

Unconversion (%)	L_E1_05	H_E1_05	L_E1_15	H_E1_15	L_E1_40	H_E1_40	L_E1_60	H_E1_60	L_E2_05	H_E2_05	L_E2_15	H_E2_15	L_E2_40	H_E2_40	L_E2_60	H_E2_60	L_E3_60	H_E3_60	L_E3_90	H_E3_90	L_B1_05	H_B1_05	L_B1_15	H_B1_15
0≤ <10	7.55	18.70	50.00	58.11	67.10	98.56	67.63	98.65	6.65	17.00	67.24	84.69	70.57	98.64	69.71	98.89	65.95	98.45	62.44	96.70	7.53	19.99	65.44	84.44
10≤ <20	7.93	8.13	12.87	18.44	0.53	0.74	0.42	0.69	7.48	8.06	3.14	9.13	0.46	0.69	0.31	0.61	0.53	0.66	0.80	1.07	8.80	8.48	3.43	8.95
20≤ <30	13.67	18.27	4.61	13.82	0.21	0.32	0.21	0.31	13.80	18.70	0.99	4.27	0.09	0.31	0.19	0.25	0.25	0.39	0.61	0.78	14.92	18.51	1.03	4.43
30≤ <40	10.52	17.29	1.27	5.12	0.10	0.15	0.12	0.14	10.58	17.02	0.33	1.07	0.11	0.13	0.03	0.10	0.18	0.22	0.41	0.48	11.68	17.12	0.37	1.23
40≤ <50	6.25	11.90	0.34	1.72	0.09	0.07	0.05	0.07	5.80	12.33	0.07	0.27	0.04	0.08	0.08	0.04	0.09	0.10	0.15	0.35	6.06	11.90	0.12	0.30
50≤ <60	4.29	15.64	0.44	2.15	0.06	0.12	0.07	0.11	4.01	16.25	0.15	0.43	0.02	0.11	0.05	0.07	0.11	0.11	0.11	0.46	4.49	14.84	0.21	0.53
60≤ <70	1.49	6.38	0.12	0.38	0.06	0.03	0.02	0.03	1.47	6.47	0.03	0.07	0.03	0.03	0.02	0.03	0.10	0.05	0.09	0.11	1.43	5.96	0.07	0.06
70≤ <80	0.29	1.81	0.07	0.05	0.03	0.01	0.02	0.01	0.31	1.96	0.03	0.02	0.03	0.00	0.01	0.00	0.02	0.01	0.02	0.03	0.32	1.52	0.03	0.01
80≤ <90	0.21	0.48	0.19	0.01	0.15	0.00	0.13	0.00	0.27	0.54	0.14	0.00	0.13	0.00	0.14	0.00	0.14	0.00	0.17	0.01	0.25	0.41	0.14	0.01
90≤ <100	1.12	0.01	0.57	0.00	0.56	0.00	0.60	0.00	0.97	0.01	0.59	0.00	0.51	0.00	0.56	0.00	0.67	0.00	0.52	0.00	0.88	0.01	0.59	0.00
100	46.68	1.39	29.52	0.20	31.11	0.01	30.72	0.00	48.67	1.65	27.28	0.04	28.00	0.01	28.89	0.01	31.97	0.00	34.68	0.01	43.65	1.25	28.57	0.04

Unconversion (%)	L_B1_40	H_B1_40	L_B1_60	H_B1_60	L_B2_60	H_B2_60	L_L1_05	H_L1_05	L_L1_15	H_L1_15	L_L1_40	H_L1_40	L_L1_60	H_L1_60	L_L2_60	H_L2_60	L_S1_05	H_S1_05	L_S1_15	H_S1_15	L_S1_40	H_S1_40	L_S1_60	H_S1_60
0≤ <10	70.36	98.15	76.11	98.64	50.64	97.98	7.21	19.71	63.52	84.73	66.36	98.47	66.35	98.30	64.67	98.37	8.47	21.58	59.63	85.06	67.53	98.58	71.32	98.53
10≤ <20	0.48	0.89	0.33	0.68	0.41	0.90	8.00	8.13	2.97	9.15	0.40	0.76	0.59	0.81	0.54	0.85	9.46	10.81	3.00	9.16	0.42	0.71	0.36	0.71
20≤ <30	0.23	0.45	0.20	0.33	0.49	0.42	13.57	18.47	0.85	4.16	0.20	0.36	0.18	0.42	0.17	0.38	13.37	21.34	0.82	3.92	0.15	0.32	0.20	0.33
30≤ <40	0.14	0.22	0.10	0.15	0.39	0.24	10.85	16.98	0.26	1.09	0.17	0.16	0.13	0.21	0.17	0.18	8.31	17.37	0.30	1.07	0.08	0.16	0.07	0.18
40≤ <50	0.07	0.10	0.06	0.07	0.19	0.18	6.10	11.96	0.15	0.29	0.08	0.08	0.05	0.10	0.05	0.08	4.15	10.99	0.10	0.23	0.10	0.07	0.04	0.09
50≤ <60	0.05	0.14	0.03	0.09	0.17	0.19	4.28	15.00	0.14	0.46	0.07	0.11	0.05	0.11	0.06	0.09	3.21	12.18	0.18	0.42	0.03	0.11	0.04	0.11
60≤ <70	0.04	0.03	0.03	0.03	0.11	0.07	1.59	6.23	0.09	0.07	0.05	0.04	0.06	0.04	0.06	0.04	1.26	4.23	0.07	0.09	0.03	0.03	0.04	0.04
70≤ <80	0.01	0.01	0.00	0.00	0.09	0.02	0.35	1.65	0.08	0.02	0.03	0.01	0.01	0.01	0.01	0.01	0.30	0.82	0.06	0.03	0.03	0.01	0.02	0.01
80≤ <90	0.12	0.00	0.13	0.00	0.39	0.01	0.31	0.46	0.16	0.00	0.17	0.00	0.15	0.00	0.18	0.00	0.33	0.20	0.23	0.01	0.15	0.00	0.19	0.00
90≤ <100	0.55	0.00	0.37	0.00	1.05	0.00	1.02	0.01	0.53	0.00	0.54	0.00	0.62	0.00	0.64	0.00	1.14	0.01	0.68	0.00	0.58	0.00	0.55	0.00
100	27.94	0.01	22.64	0.01	46.07	0.01	46.72	1.40	31.24	0.04	31.93	0.01	31.80	0.00	33.46	0.00	50.01	0.48	34.93	0.02	30.87	0.01	27.18	0.01

f **λDNA^{-mC}**

Unconversion (%)	p_E1_05	m_E1_05	p_E1_15	m_E1_15	p_E1_40	m_E1_40	p_E1_60	m_E1_60	p_E2_05	m_E2_05	p_E2_15	m_E2_15	p_E2_40	m_E2_40	p_E2_60	m_E2_60	p_E3_60	m_E3_60	p_E3_90	m_E3_90	p_B1_05	m_B1_05	p_B1_15	m_B1_15
0≤ <10	7.79	9.88	69.61	77.58	98.94	98.84	98.75	98.94	7.33	9.67	95.03	95.85	98.75	98.62	99.05	99.20	98.49	98.19	96.61	95.15	8.35	10.00	94.85	95.80
10≤ <20	14.43	22.12	22.19	16.14	0.55	0.63	0.59	0.68	15.43	21.77	3.75	2.80	0.61	0.67	0.50	0.48	0.62	0.76	1.07	1.68	15.28	21.92	3.88	2.91
20≤ <30	28.33	32.32	6.04	4.44	0.21	0.27	0.16	0.19	28.49	32.39	0.78	0.82	0.25	0.31	0.14	0.16	0.32	0.58	0.95	1.54	28.76	32.84	0.79	0.78
30≤ <40	24.83	21.33	1.28	0.98	0.12	0.12	0.12	0.06	24.52	20.80	0.16	0.34	0.14	0.11	0.07	0.11	0.20	0.20	0.72	0.79	23.98	20.87	0.21	0.24
40≤ <50	14.02	8.47	0.30	0.33	0.06	0.04	0.06	0.07	13.59	9.22	0.11	0.04	0.09	0.11	0.04	0.00	0.11	0.14	0.30	0.44	13.38	8.41	0.07	0.07
50≤ <60	7.53	4.05	0.33	0.28	0.05	0.02	0.03	0.02	7.47	4.10	0.04	0.08	0.05	0.07	0.05	0.01	0.08	0.07	0.07	0.18	7.33	4.19	0.07	0.11
60≤ <70	2.13	0.98	0.10	0.04	0.01	0.01	0.02	0.02	2.17	1.19	0.01	0.01	0.01	0.01	0.00	0.01	0.03	0.02	0.09	0.09	2.12	1.06	0.04	0.01
70≤ <80	0.45	0.26	0.03	0.00	0.01	0.00	0.01	0.01	0.48	0.32	0.00	0.00	0.00	0.00	0.00	0.00	0.00	0.00	0.00	0.00	0.41	0.21	0.01	0.00
80≤ <90	0.13	0.06	0.00	0.02	0.00	0.01	0.01	0.00	0.15	0.07	0.01	0.00	0.00	0.01	0.00	0.00	0.01	0.00	0.02	0.00	0.09	0.03	0.01	0.00
90≤ <100	0.00	0.01	0.01	0.01	0.00	0.00	0.00	0.00	0.00	0.02	0.00	0.00	0.00	0.00	0.00	0.00	0.00	0.00	0.00	0.00	0.00	0.01	0.00	0.00
100	0.36	0.51	0.13	0.19	0.04	0.06	0.24	0.02	0.37	0.45	0.12	0.06	0.10	0.08	0.15	0.02	0.15	0.04	0.16	0.13	0.30	0.45	0.07	0.07

Unconversion (%)	p_B1_40	m_B1_40	p_B1_60	m_B1_60	p_B2_60	m_B2_60	p_L1_05	m_L1_05	p_L1_15	m_L1_15	p_L1_40	m_L1_40	p_L1_60	m_L1_60	p_L2_60	m_L2_60	p_S1_05	m_S1_05	p_S1_15	m_S1_15	p_S1_40	m_S1_40	p_S1_60	m_S1_60
0≤ <10	98.67	98.87	98.83	99.03	99.04	98.96	7.59	9.18	94.96	95.90	98.82	98.99	98.93	98.80	99.03	99.11	22.76	26.12	90.53	90.87	93.63	93.31	92.82	93.12
10≤ <20	0.73	0.62	0.53	0.54	0.58	0.47	14.44	21.01	3.76	2.81	0.60	0.47	0.64	0.53	0.52	0.45	18.81	27.40	4.26	4.14	2.75	2.39	2.27	2.94
20≤ <30	0.29	0.22	0.23	0.20	0.14	0.32	28.48	32.78	0.70	0.68	0.18	0.29	0.15	0.37	0.15	0.17	28.37	27.40	1.90	2.55	1.49	1.65	1.89	2.08
30≤ <40	0.10	0.13	0.09	0.12	0.08	0.11	24.20	21.45	0.19	0.26	0.12	0.09	0.08	0.16	0.08	0.10	16.29	11.91	1.65	1.25	0.46	1.57	1.35	0.90
40≤ <50	0.05	0.13	0.06	0.03	0.09	0.06	13.99	9.13	0.10	0.09	0.06	0.04	0.05	0.05	0.04	0.07	8.48	4.50	0.66	0.34	0.46	0.50	0.64	0.72
50≤ <60	0.05	0.01	0.05	0.03	0.02	0.00	7.99	4.48	0.10	0.09	0.08	0.06	0.03	0.03	0.05	0.04	3.86	1.88	0.37	0.68	0.80	0.41	0.66	0.11
60≤ <70	0.02	0.00	0.02	0.01	0.00	0.04	2.34	1.13	0.05	0.02	0.02	0.00	0.01	0.01	0.03	0.01	0.90	0.61	0.29	0.06	0.23	0.08	0.26	0.07
70≤ <80	0.00	0.00	0.00	0.00	0.00	0.00	0.51	0.29	0.00	0.00	0.02	0.01	0.00	0.01	0.00	0.00	0.18	0.06	0.04	0.06	0.00	0.00	0.00	0.04
80≤ <90	0.00	0.00	0.01	0.00	0.00	0.00	0.11	0.08	0.00	0.01	0.01	0.00	0.01	0.00	0.00	0.00	0.13	0.00	0.00	0.00	0.00	0.00	0.00	0.00
90≤ <100	0.00	0.00	0.00	0.00	0.00	0.00	0.01	0.01	0.00	0.00	0.00	0.00	0.00	0.00	0.00	0.00	0.00	0.00	0.00	0.00	0.00	0.00	0.00	0.00
100	0.07	0.02	0.17	0.03	0.06	0.04	0.33	0.46	0.14	0.11	0.08	0.06	0.10	0.04	0.11	0.05	0.22	0.12	0.29	0.06	0.17	0.08	0.12	0.04

100.00

0.00

(%)

Supplementary Figure S2

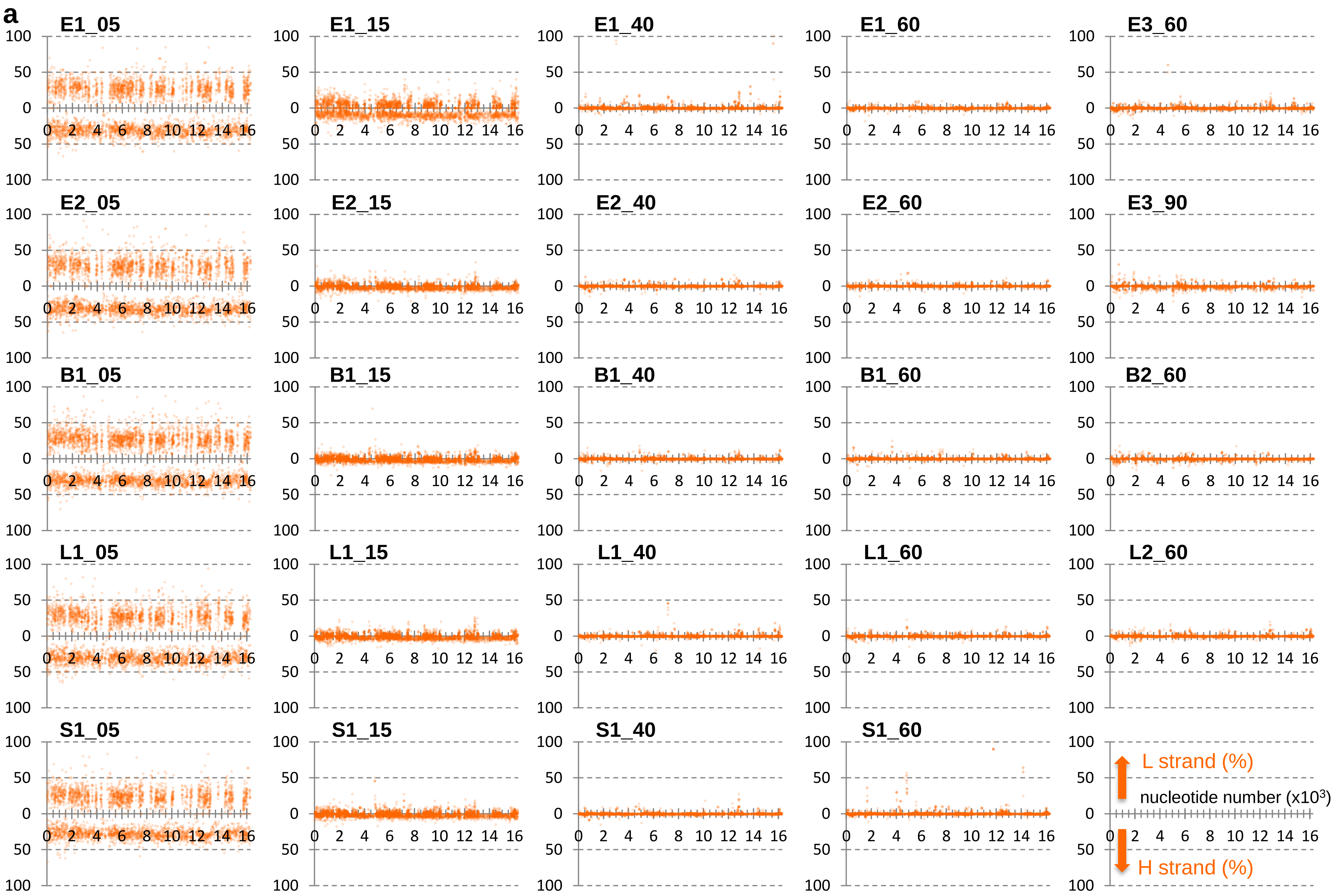

Supplementary Figure S3

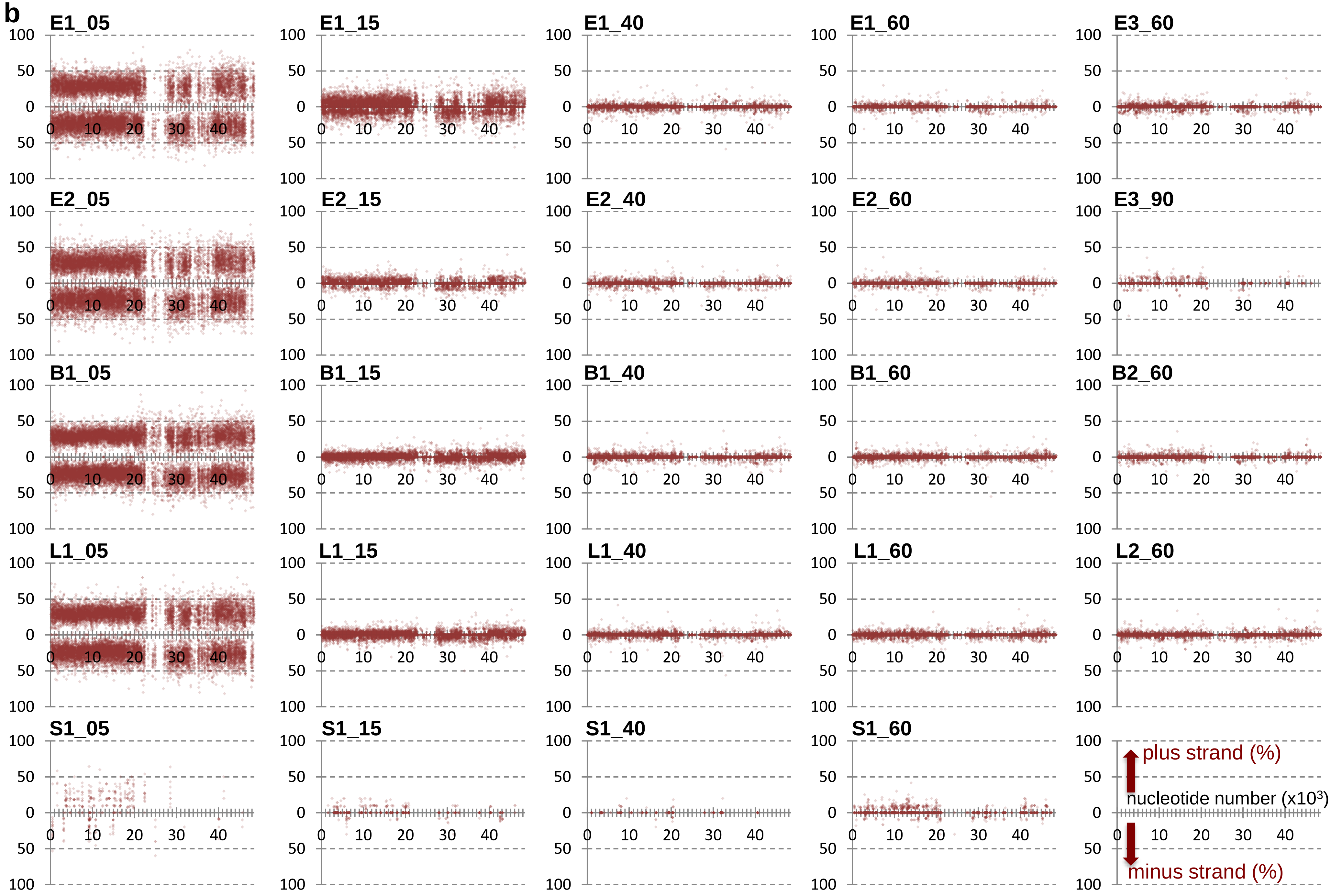

Supplementary Figure S3

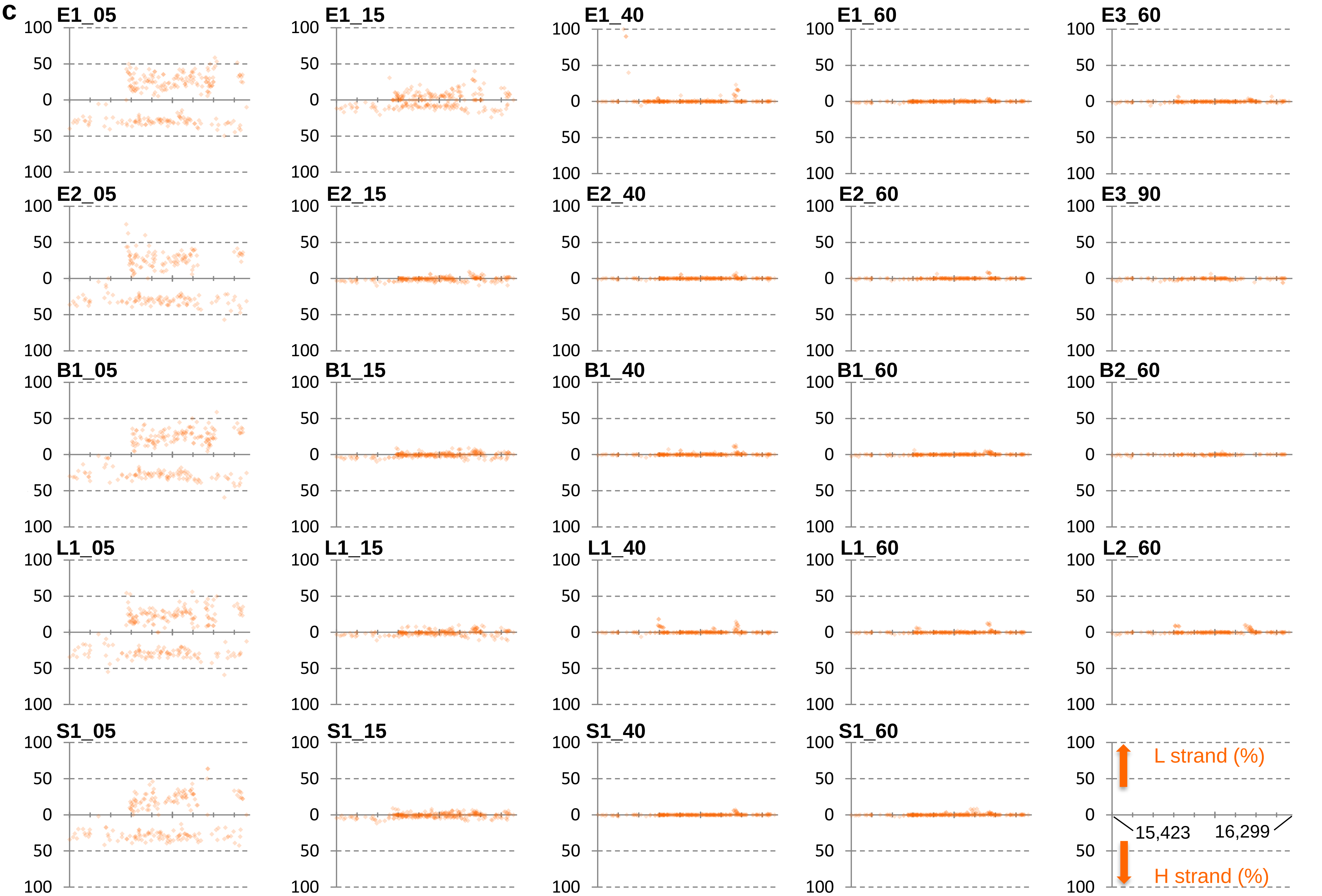

Supplementary Figure S3

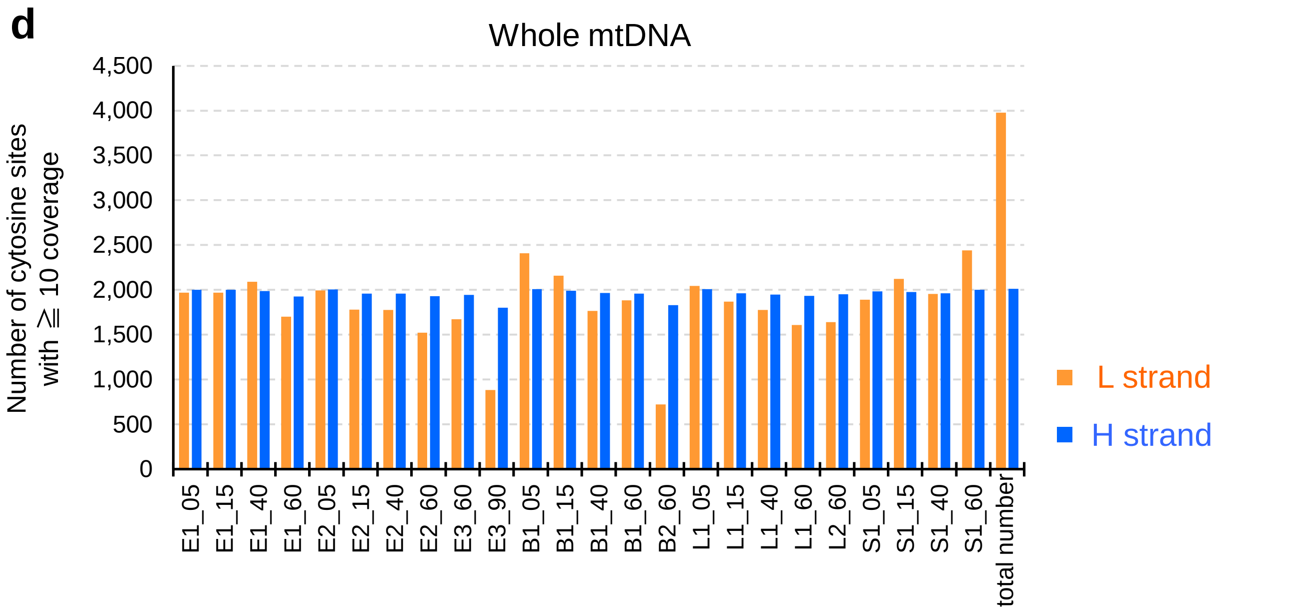

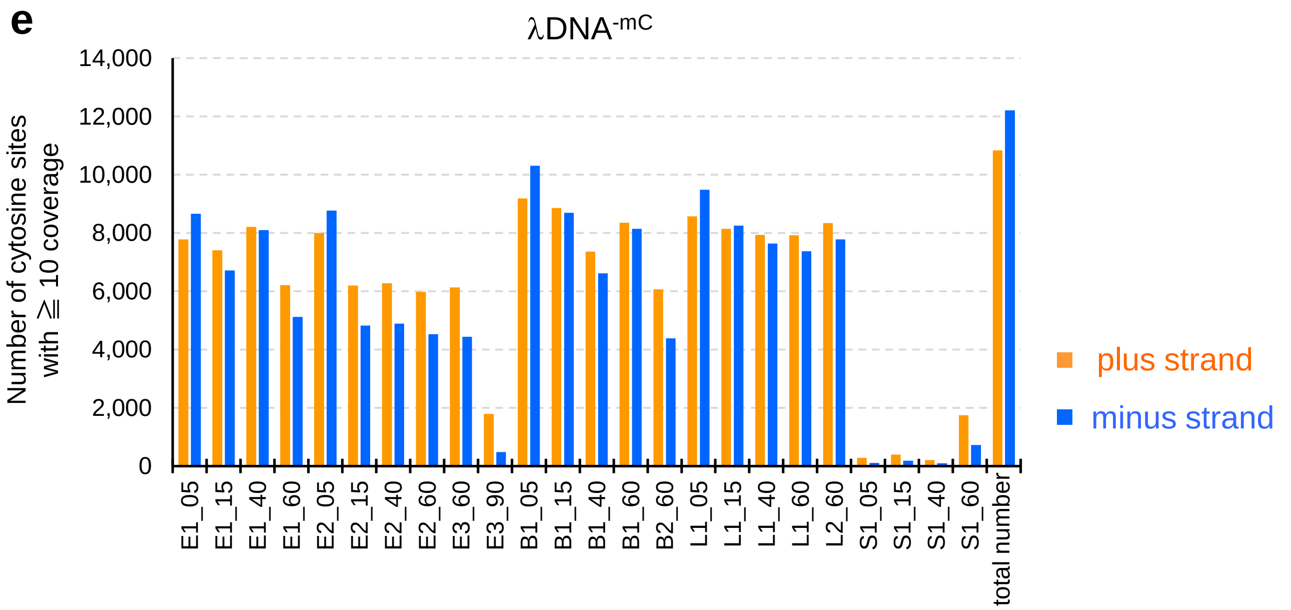

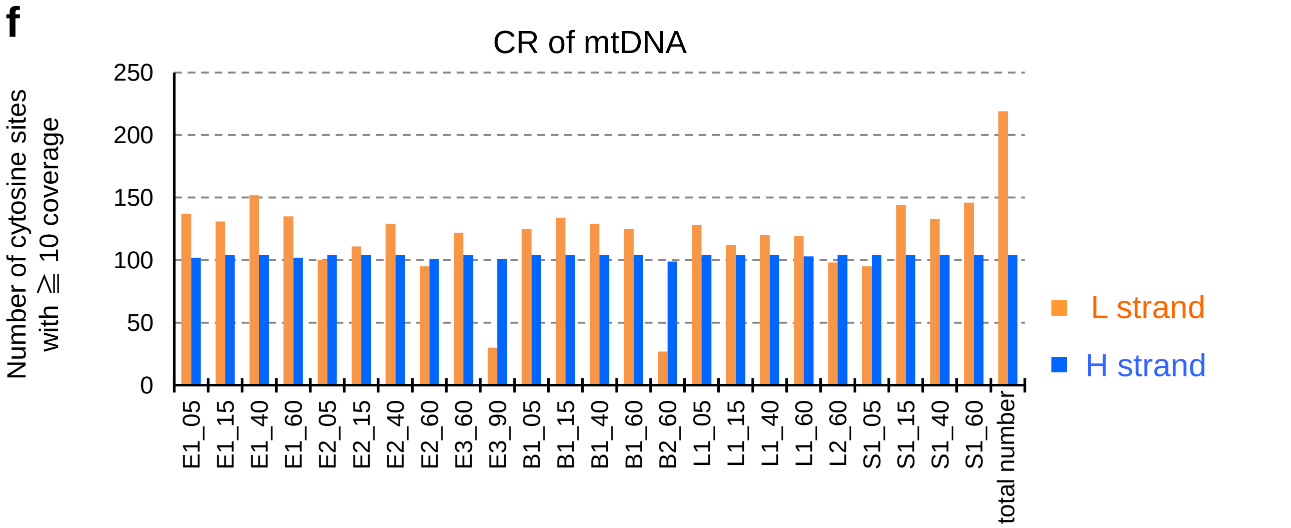

Supplementary Figure S3

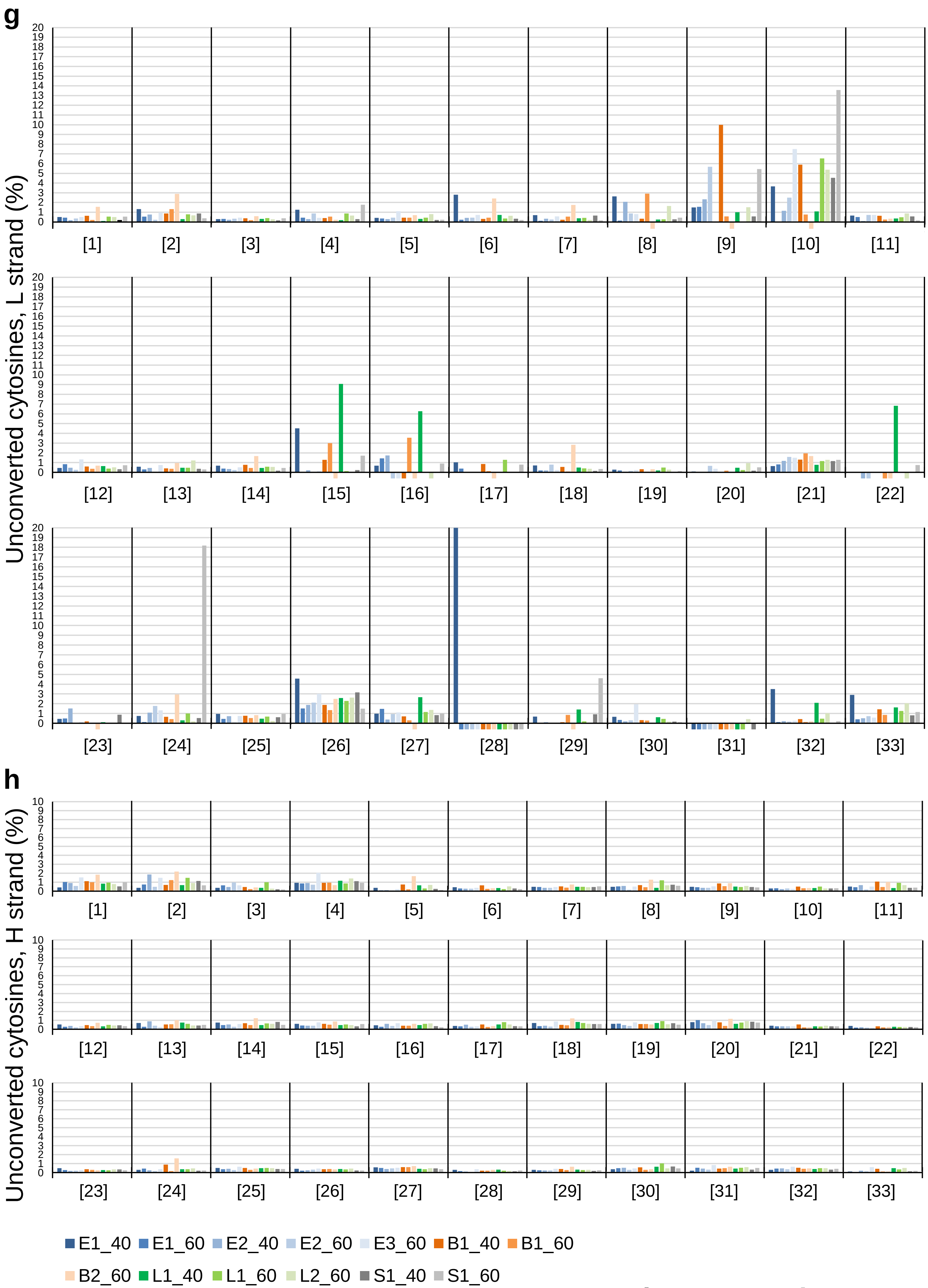

Supplementary Figure S3

i

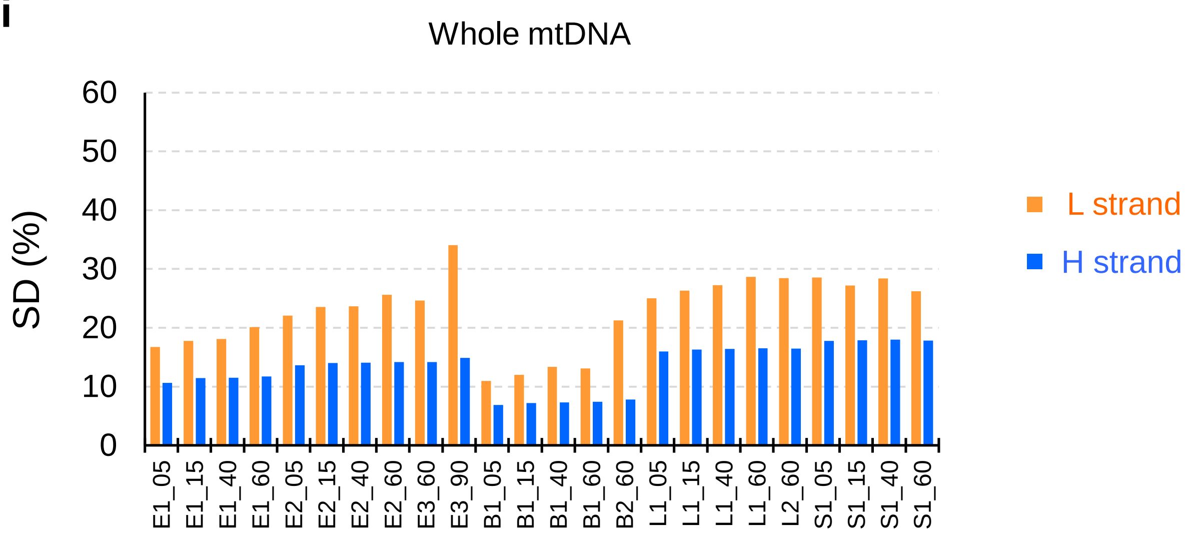

j

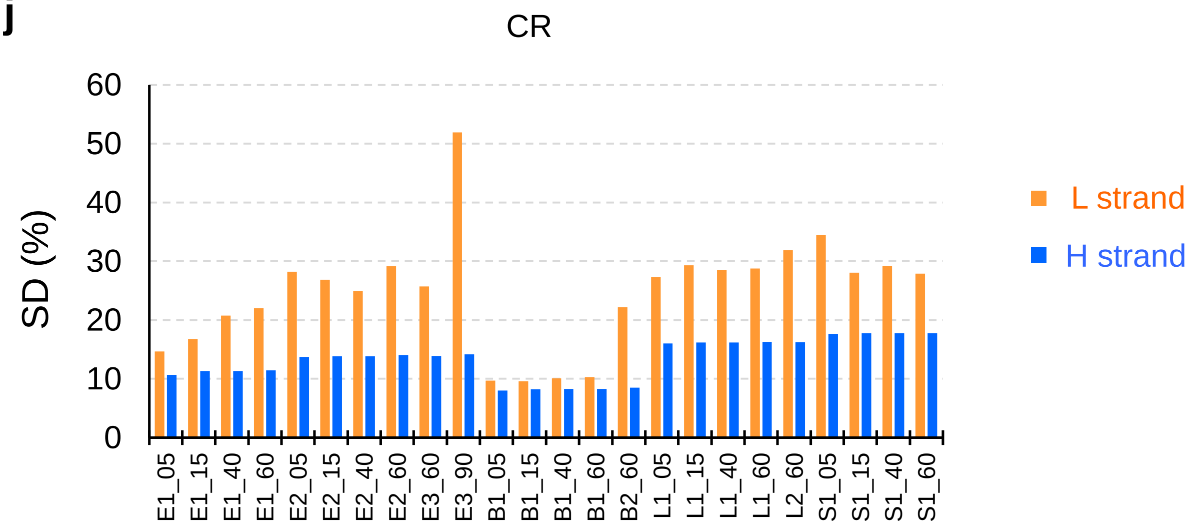

Supplementary Figure S3

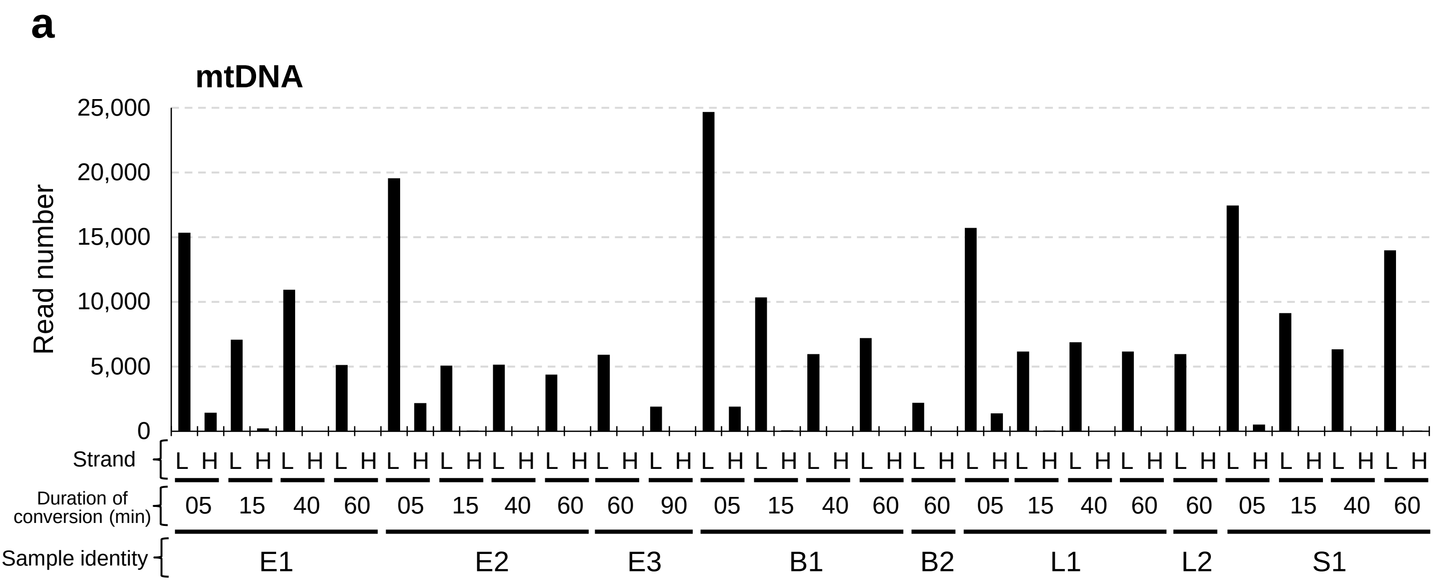

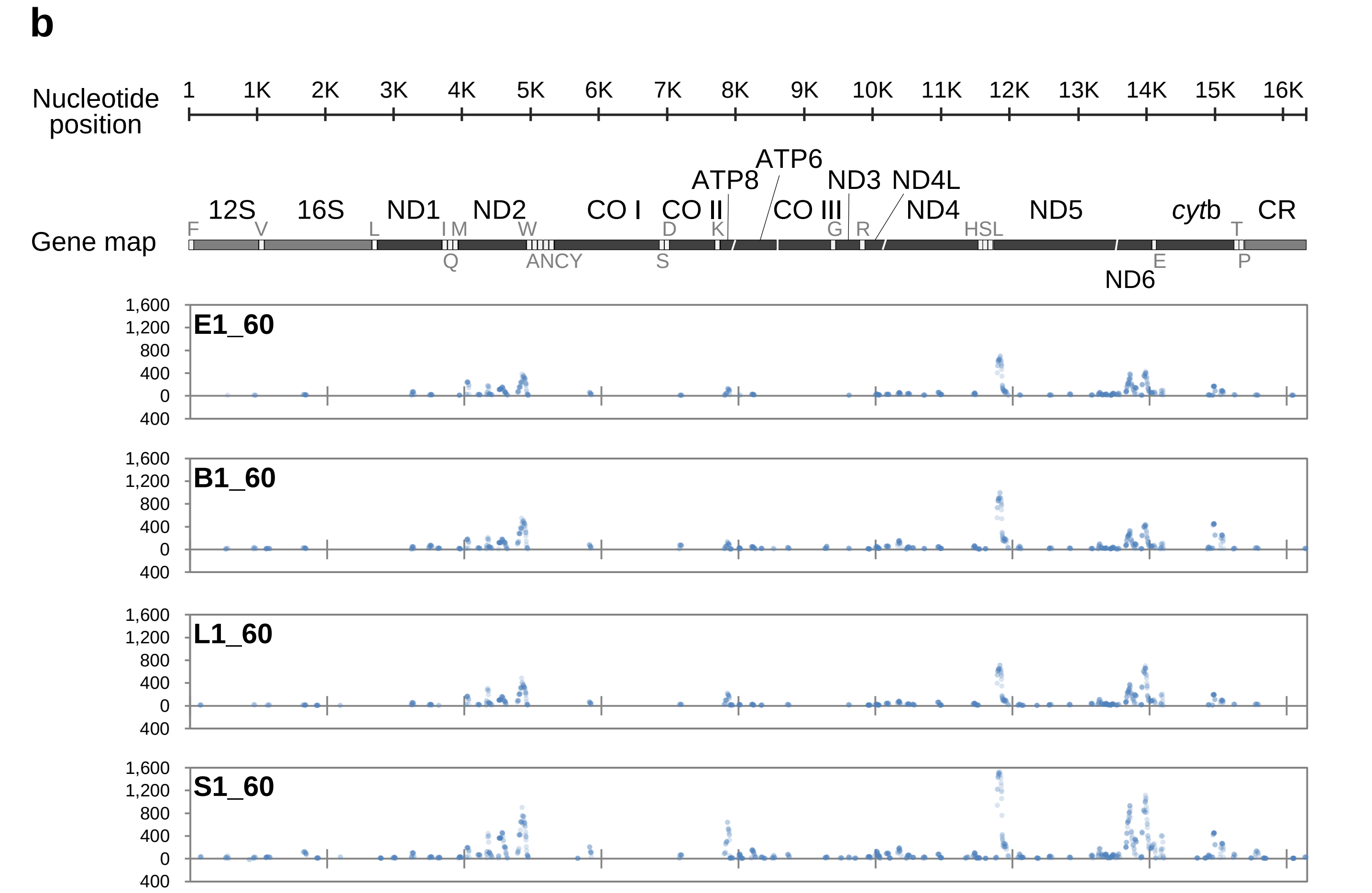

Supplementary Figure S4

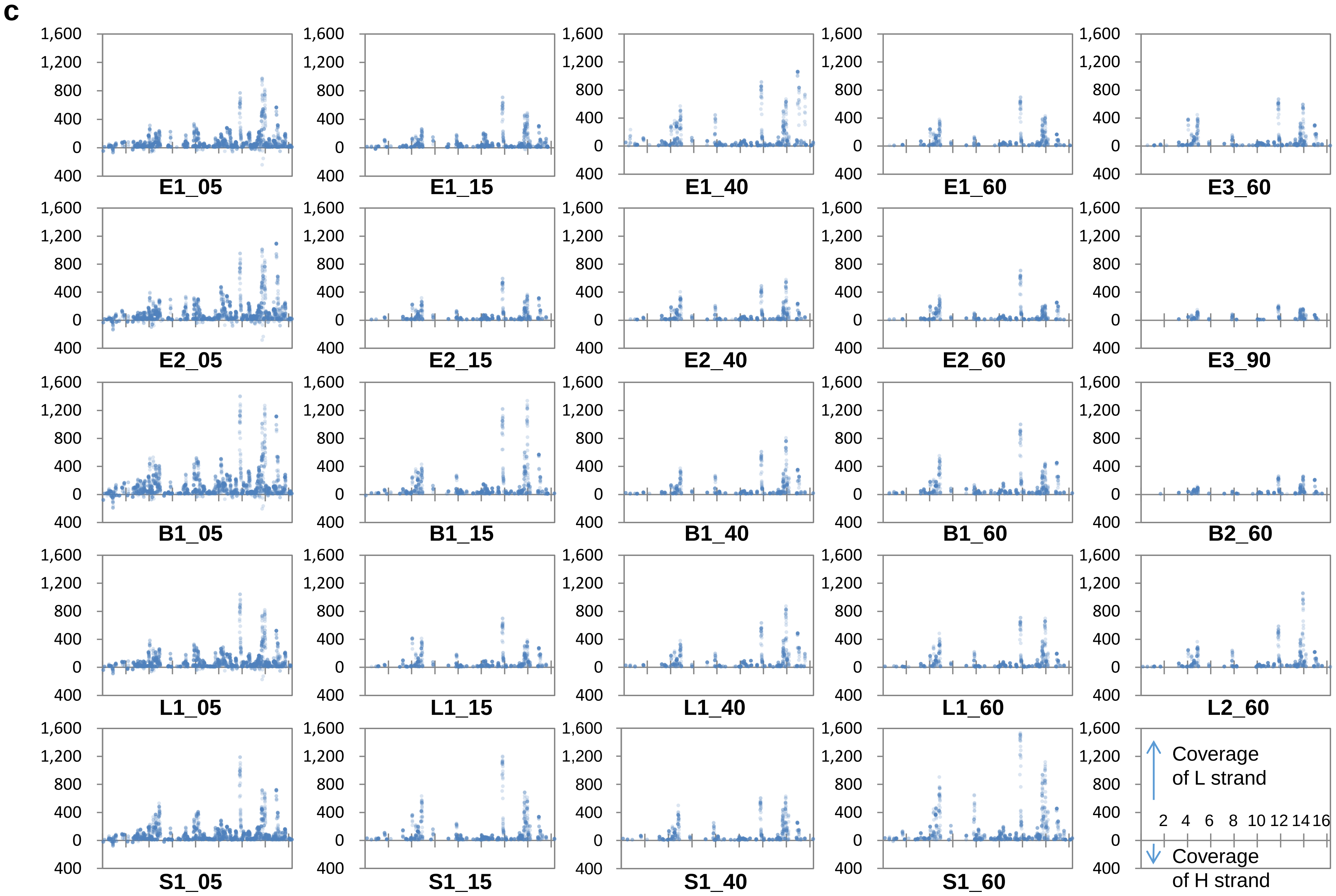

Supplementary Figure S4

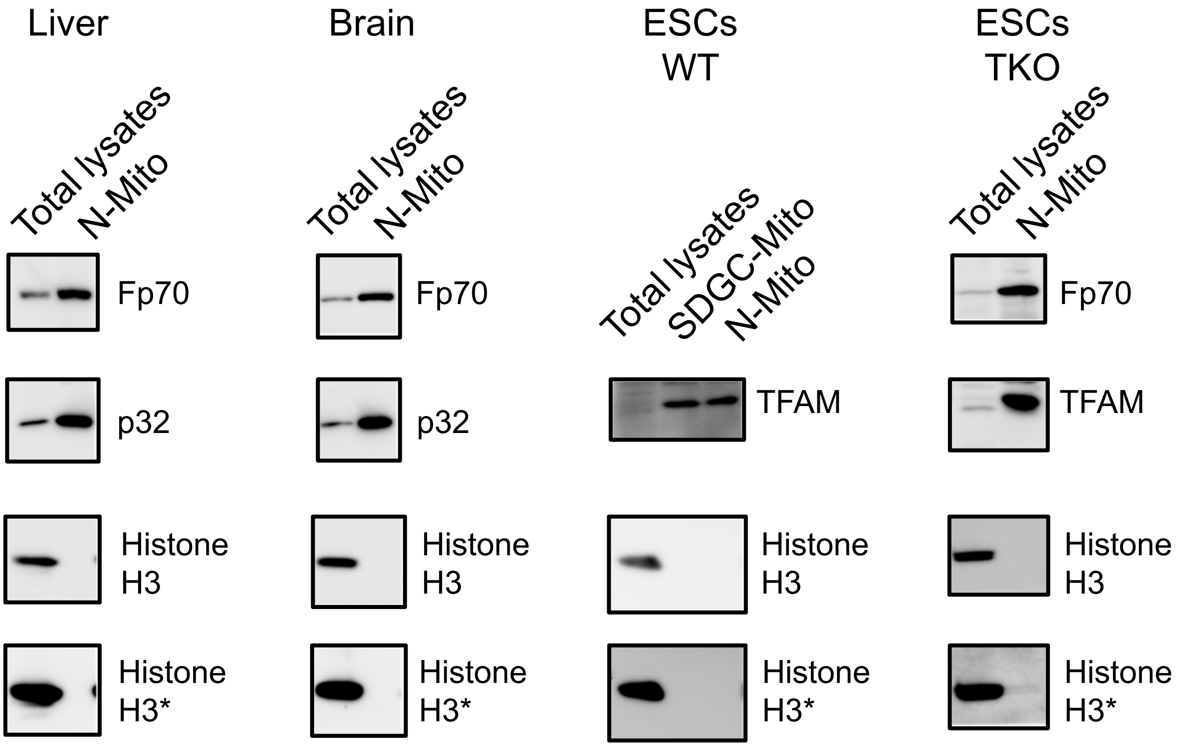

Supplementary Figure S5

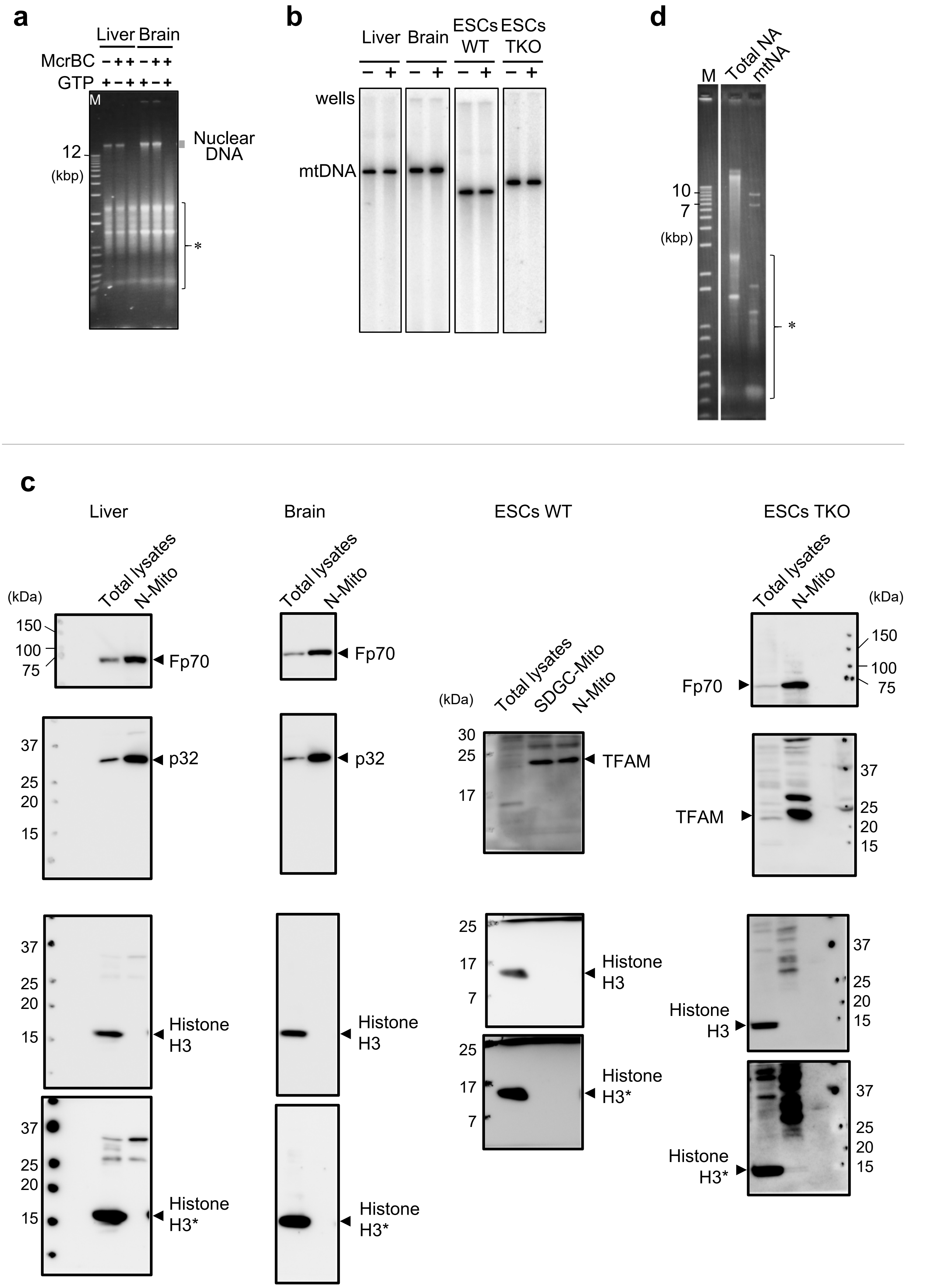

Supplementary Figure S6

Supplementary Table S1. Analysis of published WGBS metadata

(1)~(8) are shown in Supplementary Fig. S			(1)	(2)	(3)	(4)	(5)	(6)	(7)	(8)														
species			mouse	mouse	mouse	mouse	mouse	mouse	mouse	mouse	mouse	mouse	mouse	mouse	mouse	mouse	mouse	mouse	mouse	mouse	human	human	human	human
sample			liver[1]	cerebellum[1]	cortex[1]	heart[1]	kidney[1]	ESC (2i/LIF)[2]	ESC 1 (Serum/LIF)[2]	ESC 2 (Serum/LIF)[3]	ESC [tko][4]	Non- growing oocyte[5]	Germinal vesicle oocyte[5]	Dnmt3a KO oocyte[5]	Dnmt3b KO oocyte[5]	Dnmt1 KO oocyte[5]	Dnmt3l KO oocyte[5]	Oocyte[6]	Inner cell mass[6]	Male PGC (E13.5)[7]	fibroblast-derived iPS cells (clone 6_9)[8]	ovary[8]	ESC[9]	New born-derived foreskin fibroblast[10]
Data ID			SRX209455	SRX209449	SRX209451	SRX209452	SRX209454	SRX210604,SRX210603,SRX210602	SRX210599,SRX210600,SRX210601	GSE30202	SRX204133	DRX001591,DRX001592	DRX001583, DRX001584	DRX001586	DRX001587, DRX001590	DRX001589, DRX001585	DRX001588	GSM1386019	GSM1386023	DRX001803~DRX001807	SRX056692	SRR1045705	SRX006239,SRX006240	GSM491351
library			MethylC single	MethylC single	MethylC single	MethylC single	MethylC single	MethylC single	MethylC single	MethylC single	MethylC paired	PBAT single	PBAT single	PBAT single	PBAT single	PBAT single	PBAT single	MethylC paired	MethylC paired	PBAT paired	MethylC single	MethylC single	MethylC single	MethylC paired
			Average levels of unconverted cytosines in percentages (depth>=10)																					
Mitochondria	CG	L strand	0.51	0.89	0.75	0.45	0.57	17.68	12.14	4.82	2.88	1.8	7.77	4.44	7.51	7.23	2.33	2.34	0.87	0.81	0.41	0.33	2.15	0.38
		H strand	0.49	0.53	0.4	0.43	0.48	1.2	1.08	1.09	0.54	0.79	3.39	1.5	3.02	2.69	0.84	0.4	0.88	0.87	0.36	0.38	0.46	0.09
	CHG	L strand	0.56	0.94	1.08	0.44	0.7	21.55	13.49	7.98	2.92	1.79	7.92	4.41	7.6	7.27	2.54	2.82	0.95	0.79	0.39	0.32	2.28	0.2
		H strand	0.44	0.51	0.38	0.37	0.43	1.27	0.83	1.05	0.47	0.81	3.58	1.55	3.21	2.79	0.9	0.46	0.9	0.8	0.39	0.38	0.39	0.07
	CHH	L strand	0.57	1.19	1.41	0.44	0.96	26.84	17.69	9.97	4.18	2.25	12.4	7.66	12.19	11.39	4.38	7.66	1	0.94	0.48	0.31	2.67	0.35
		H strand	0.43	0.64	0.41	0.42	0.51	2.14	1.87	1.27	0.58	0.82	3.17	1.41	2.79	2.48	0.83	0.5	0.92	0.87	0.39	0.35	0.43	0.07
Nucleus	CG	Top	78.13	80.03	79.51	75.79	79.29	25.65	68.87	78.74	3.16	3.2	39.32	6.55	39.3	38.27	3.31	58.34	21.72	3.51	86.21	65.42	84.87	54.27
		Bottom	78.1	80.06	79.53	75.78	79.08	25.62	68.78	78.69	1.6	3.33	39.43	6.57	39.38	38.43	3.33	58.3	21.74	3.57	86.18	64.72	84.9	54.05
	CHG	Top	0.5	0.94	1.28	0.57	0.56	0.81	1.12	2.09	1.64	0.54	3.68	0.68	3.83	4.36	0.43	3.45	1	0.73	2.63	0.49	2.94	0.12
		Bottom	0.5	0.94	1.28	0.57	0.56	0.81	1.12	2.1	1.02	0.55	3.69	0.68	3.83	4.35	0.42	3.46	1.01	0.73	2.64	0.49	2.94	0.12
	CHH	Top	0.51	1.09	1.51	0.61	0.6	0.82	0.81	1.66	3.11	0.56	3.12	0.62	3.21	3.54	0.43	3.29	0.97	0.72	1.06	0.54	1.2	0.13
		Bottom	0.51	1.1	1.51	0.61	0.6	0.82	0.81	1.66	1.33	0.57	3.12	0.62	3.22	3.54	0.43	3.29	0.97	0.73	1.06	0.52	1.21	0.12

			Analysed site (depth>=10)																					
Mitochondria	CG	L strand	206	171	206	207	177	147	133	187	202	169	207	207	207	207	207	227	233	179	305	377	288	213
		H strand	217	216	217	218	216	215	215	215	222	213	217	217	217	217	217	242	239	214	399	399	374	433
	CHG	L strand	273	213	272	276	217	171	149	246	262	218	272	272	272	271	271	298	308	225	356	450	344	236
		H strand	275	275	280	281	275	275	275	275	290	272	284	284	284	284	284	320	318	271	415	415	397	457
	CHH	L strand	2,277	1,436	2,229	2,293	1,439	1,064	927	1,754	1,880	1,691	2,302	2,299	2,302	2,301	2,294	2,511	2610	1796	2,417	3,738	2,295	1,160
		H strand	998	996	999	1,009	996	986	988	989	1,034	984	1,028	1,027	1,027	1,027	1,027	1,146	1134	984	1,137	1,136	1,079	1,259
Nucleus	CG	Top	565,242	1,495,168	1,586,934	2,508,201	205,110	4,503,615	4,047,727	197,145	3,709	66,631	3,818,400	644,287	4,589,785	911,554	454,107	9,513,590	11232403	77391	2,991,472	10,497	1,119,526	1,073,472
		Bottom	567,378	1,496,838	1,585,137	2,505,342	203,968	4,506,973	4,050,441	197,804	3,652	67,737	3,821,966	643,127	4,590,984	913,434	452,871	9,516,453	11230846	78349	2,976,664	9,889	1,116,865	1,067,404
	CHG	Top	3,227,186	7,633,512	8,472,431	13,067,861	1,187,367	19,180,208	16,940,234	1,127,320	13,839	387,558	20,769,775	3,718,459	24,961,978	5,260,779	2,486,959	46,410,882	61676035	381022	15,063,189	43,948	5,568,542	4,170,761
		Bottom	3,223,895	7,628,849	8,468,716	13,056,193	1,180,201	19,156,211	16,916,124	1,127,915	13,831	387,849	20,767,149	3,715,200	24,953,954	5,259,783	2,486,918	46,368,993	61678579	380173	14,999,110	44,328	5,574,127	4,163,643
	CHH	Top	11,586,341	18,194,450	22,380,171	42,290,285	3,310,774	37,573,305	32,547,882	2,927,723	32,283	1,112,349	63,828,146	10,991,705	77,409,665	15,401,916	7,497,939	121,844,148	194887184	1157975	37,075,402	182,775	14,023,795	6,987,315
		Bottom	11,566,811	18,184,603	22,371,505	42,256,084	3,296,440	37,548,002	32,525,795	2,921,073	30,867	1,115,234	63,816,049	10,995,376	77,389,349	15,415,189	7,508,102	121,760,447	194975420	1160110	36,922,498	180,444	14,030,899	6,969,333

From published WGBS metadata, reads that were derived from mtDNA and nuclear DNA were aligned as described inMethods. Average levels of methylated/unconverted cytosines in CG, CHG and CHH sequences (H = A, G and T) with distinctions of strands in mtDNA (mitochondria) and nuclear DNA (nucleus) are presented. Numbers of analysed sites that were used to obtain averages are also shown.

Supplementary Table S2.

Conversion rate of mtDNA with 1 h bisulfite conversion reaction (%)									
mtDNA	all CN	all CG	all CH	L CN	L CG	L CH	H CN	H CG	H CH
ESC mtDNA (n = 3)	**99.5**	99.5	99.5	99.4 (99.6)	99.5	99.4	99.6 (99.5)	99.5	99.6
Brain mtDNA (n = 2)	**99.3**	99.4	99.3	99.2 (99.7)	99.2	99.2	99.4 (99.6)	99.5	99.4
Liver mtDNA (n = 2)	**99.4**	99.5	99.4	99.4 (98.8)	99.5	99.3	99.4 (99.5)	99.5	99.4
Synthetic mtDNA	**99.1**	99.6	99.1	98.7 (99.4)	99.5	98.7	99.6 (99.6)	99.6	99.6

Conversion rate of λDNA^{-mC} with 1 h bisulfite conversion reaction (%)									
λDNA^{-mC} mixed in	all CN	all CG	all CH	plus CN	plus CG	plus CH	minus CN	minus CG	minus CH
ESC samples (n = 3)	**99.6**	99.6	99.6	99.6	99.6	99.6	99.6	99.7	99.6
Brain samples (n = 2)	**98.6**	98.8	98.4	98.6	98.6	98.6	98.6	98.6	98.4
Liver samples (n = 2)	**99.6**	99.6	99.6	99.6	99.6	99.6	99.7	99.6	99.7
Synthetic mtDNA	**98.3**	98.4	98.3	98.2	98.3	98.1	98.6	98.5	98.7

Numbers shown for ESC, brain and liver mtDNA are means of cytosine conversion rates obtained from data of three (ESCs) or two (brain and liver) independently prepared samples. Numbers for synthetic mtDNA are cytosine conversion rates obtained from data of a single preparation. Numbers in parentheses indicate conversion rates in the CR of mtDNA (nt15,423-16,299).
